# Young Human Cholinergic Neurons Respond to Physiological Regulators and Improve Cognitive Symptoms in an Animal Model of Alzheimer’s Disease

**DOI:** 10.3389/fncel.2017.00339

**Published:** 2017-10-27

**Authors:** Annamaria Morelli, Erica Sarchielli, Giulia Guarnieri, Elisabetta Coppi, Daniela Pantano, Paolo Comeglio, Pamela Nardiello, Anna M. Pugliese, Lara Ballerini, Rosanna Matucci, Stefano Ambrosini, Giuseppe Castronovo, Rosa Valente, Benedetta Mazzanti, Sandra Bucciantini, Mario Maggi, Fiorella Casamenti, Pasquale Gallina, Gabriella B. Vannelli

**Affiliations:** ^1^Section of Human Anatomy and Histology, Department of Experimental and Clinical Medicine, University of Florence, Florence, Italy; ^2^Department of Neuroscience, Psychology, Drug Research and Child Health, Division of Pharmacology and Toxicology, University of Florence, Florence, Italy; ^3^Sexual Medicine and Andrology Unit, Department of Experimental and Clinical Biomedical Sciences “Mario Serio”, University of Florence, Florence, Italy; ^4^Cell Therapy and Transfusion Medicine Unit, Department of Experimental and Clinical Medicine, University of Florence, Florence, Italy; ^5^Department of Experimental and Clinical Biomedical Sciences “Mario Serio”, Section of Clinical Physiopathology, Florence, Italy; ^6^Careggi University Hospital, Florence, Italy; ^7^Neurosurgery School of Tuscany, Department of Surgery and Translational Medicine, University of Florence, Florence, Italy

**Keywords:** nucleus basalis of Meynert, cell-based therapy, NGF, estrogen receptors, primary cilium, cholinergic receptors

## Abstract

The degeneration of cholinergic neurons of the nucleus basalis of Meynert (NBM) in the basal forebrain (BF) is associated to the cognitive decline of Alzheimer’s disease (AD) patients. To date no resolutive therapies exist. Cell-based replacement therapy is a strategy currently under consideration, although the mechanisms underlying the generation of stem cell-derived NBM cholinergic neurons able of functional integration remain to be clarified. Since fetal brain is an optimal source of neuronal cells committed towards a specific phenotype, this study is aimed at isolating cholinergic neurons from the human fetal NBM (hfNBMs) in order to study their phenotypic, maturational and functional properties. Extensive characterization confirmed the cholinergic identity of hfNBMs, including positivity for specific markers (such as choline acetyltransferase) and acetylcholine (Ach) release. Electrophysiological measurements provided the functional validation of hfNBM cells, which exhibited the activation of peculiar sodium (I_Na_) and potassium (I_K_) currents, as well as the presence of functional cholinergic receptors. Accordingly, hfNBMs express both nicotinic and muscarinic receptors, which were activated by Ach. The hfNBMs cholinergic phenotype was regulated by the nerve growth factor (NGF), through the activation of the high-affinity NGF receptor TrkA, as well as by 17-β-estradiol through a peculiar recruitment of its own receptors. When intravenously administered in NBM-lesioned rats, hfNBMs determined a significant improvement in memory functions. Histological examination of brain sections showed that hfNBMs (labeled with PKH26 fluorescent dye prior to administration) reached the damaged brain areas. The study provides a useful model to study the ontogenetic mechanisms regulating the development and maintenance of the human brain cholinergic system and to assess new lines of research, including disease modeling, drug discovery and cell-based therapy for AD.

## Introduction

The basal forebrain (BF) region is a broad topographic term describing a heterogeneous set of cellular structures on the medial and ventral cerebral hemisphere. It comprises the nucleus basalis of Meynert (NBM), the vertical and horizontal diagonal bands of Broca and the medial septal nucleus (Mesulam, 2013). This complex region contains magnocellular neurons that provide the major cholinergic projections to the cerebral cortex, hippocampus and amygdala. The BF cholinergic system has been implicated in the modulation of an ever-expanding set of behavioral states, including attention, memory and learning (Ferreira-Vieira et al., 2016). Importantly, the degeneration and loss of cholinergic neurons within the BF, especially in the NBM, represents a pathological correlate of the well-documented cholinergic derangement in Alzheimer’s disease (AD) patients (Kilimann et al., 2014).

Despite its implications as pathological substrate for cholinergic deficiency in AD brain, very little is known about the developmental properties of the NBM neuronal contingent during embryogenesis. As we move towards an era of stem cell-based treatments for several pathologies, including neurodegenerative disorders, the use of cholinergic cell-based transplantation approaches to treat AD should be considered. However, extensive studies are needed to identify the mechanisms underlying the generation of functionally integrated stem cell-derived NBM cholinergic neurons. Since fetal brain is an optimal resource to study developmental processes of neurons already committed towards a specific phenotype, the aim of the present study is the isolation and characterization of human cholinergic neurons from the fetal NBM.

Studies in animal models after fetal tissue transplantation have demonstrated that neuronal replacement and partial reconstruction of damaged neuronal circuitry is possible (Björklund and Lindvall, 2000). There is also evidence from clinical trials that fetal graft in the diseased human brain can lead to symptomatic relief (Paganini et al., 2014). Human neurological disorders such as AD, Parkinson’s disease (PD), Huntington’s disease, stroke or spinal cord injury are caused by the loss of neurons and glial cells in the brain or spinal cord. In each of them a different spectrum of cell types is affected; therefore, before clinical trials are initiated, we need to know much more about how to control cell proliferation and differentiation into specific phenotypes, promote their integration into existing neural and synaptic circuits, and determine functional recovery in animal models closely resembling the human disease. Recently, Grealish et al. (2014) have provided preclinical evidence that the potency of the human embryonic stem cell-derived dopaminergic neurons, with respect to functional efficacy and long-distance targeted reinnervation, is comparable to that of fetal ventral mesencephalon cells, which provides important support for their therapeutic potential and use for cell replacement therapy in PD patients.

The NBM precursor neurons were reported to originate in the telencephalic subpallium (Xu et al., 2008). A new pallial origin of these neurons has been also described (Pombero et al., 2011). These differences in the BF cholinergic system ontogenesis might explain their different connection patterns (cortex vs. hippocampus) and functions in cognitive processes, as well as their specific responses to pathological stimuli. The data on the development and anatomical organization of the NBM in the human fetal brain revealed early maturation. The earliest signs of the acetylcholinesterase (AchE) activity, indicative of acetylcholine (Ach) release, appear as early as 9 weeks of gestation (w.g.) in the area where the prospective basal nucleus develops and the first AChE-reactive bundles form between this age and 10.5 w.g. (Kostović, 1986). At 12–15 weeks, the positive cell group appear and the basal nucleus can be separated into several subdivisions. Innervation of the developing cortex and subcortical areas by AchE-reactive fibers occurs between 16 w.g. and 28 w.g. An overall high degree of structural and chemical complexity is early achieved in the NBM of human fetal brain (Kračun and Rösner, 1986), suggesting that this brain area plays an important role in the earliest cortical connections due to its specific functional entity. Of great importance for the definition of the NBM within the human brain was the demonstration that this nucleus increases both in its relative and absolute size and differentiation with increasing cerebralization (Gorry, 1963).

In this work, we isolated neurons from NBM of 12 week old human fetuses, which is the proper time during embryogenesis when the commitment into differentiation programs for BF nuclear complex is well defined (Kostović, 1986). Although retaining growth and differentiation potentials, an extensive phenotypical and functional characterization demonstrated that, in the chosen “developmental window”, these neurons possess a clear BF cholinergic identity, which may be importantly induced and maintained by physiological factors, such as nerve growth factor (NGF) and estrogens. More importantly, when intravenously injected into NBM-lesioned rats these cells reached the damaged brain area and ameliorated cognitive deficiencies typical of AD. These results provide the first characterization of human fetal BF cholinergic neurons and show their potential as a useful resource in the research field of cognitive disorders.

## Materials and Methods

Further information can be found in Supplementary Material.

### Cell Culture

The use of human fetal tissue for research purposes was approved by the National Ethics Committee and the local ethic committee for investigation in Humans of the University of Florence (Permit Number: 678304). Human fetuses biopsies were obtained from therapeutic medical abortions after women approved and signed the informed consent document, as already reported (Gallina et al., 2008). NBM tissue was dissected from two female 12-weeks old human fetuses and incubated with 1 mg/ml collagenase type IV (Sigma-Aldrich Corp., St. Louis, MO, USA). The cell suspensions were mechanically dispersed by pipetting and cultured in Coon’s modified Ham’s F12 medium (Euroclone, Milan, Italy) supplemented with 10% FBS (Hyclone, Logan, UT, USA). Cells were used within the 26th passage.

### Quantitative Real-Time RT-PCR

Isolation of total RNA and cDNA synthesis were performed using the “RNeasy Micro kit” (Qiagen, Hilden, Germany) and the iScript™ cDNA Synthesis Kit (Bio-Rad Laboratories, Hercules, CA, USA). For some genes, quantitative real time PCR (qRT-PCR) was performed according to the fluorescent TaqMan methodology, as previously described (Morelli et al., 2008) Primers and probes for the target genes were predeveloped assays (Life Technologies, Carlsbad, CA, USA) as listed in Supplementary Table S1. For the remaining genes, qRT-PCR was performed using SsoFast™ EvaGreen^®^ Supermix (Bio-Rad Laboratories) as previously described (Morelli et al., 2013). Specific primers sequences for the target genes are reported in Supplementary Table S2. The 18S ribosomal RNA subunit was chosen as the housekeeping gene for relative quantification. Data analysis was based on the comparative threshold cycle (Ct) using the 2^–∆∆Ct^ method and carried out with the MyIQ2™ Two-Color Real-Time PCR Detection System (Bio-Rad Laboratories).

### Immunofluorescence

The analysis was performed as previously described (Sarchielli et al., 2014) using the following primary antibodies: anti-ChAT polyclonal antibody (pAb; 1:200; Millipore, Temecula, CA, USA), anti-VAchT pAb (1:1000), anti-acetylated α-tubulin mAb (1:500) from Sigma-Aldrich Corp., St. Louis, MO, USA; anti-GFAP monoclonal antibody (mAb; 1:100), anti-α tubulin mAb (1:2000), anti-ERα mAb (1:50), anti-ERβ mAb (1:50) from Santa Cruz Biotechnology (Santa Cruz, CA, USA); anti-GPR30 pAb (1:40, Abcam, Cambridge, UK), followed by Alexa Fluor 488 or 568 goat anti-rabbit or Alexa Fluor 488 goat anti-mouse (1:200, Molecular Probes, Eugene, Oregon), as appropriate. For α-tubulin and acetylated α-tubulin staining, cells were cultured in serum/phenol red-free condition and treated with NGF (100 ng/ml), E2 (10 nM) or G1 (100 nM) for 24/48 h in presence or absence of the receptor inhibitors K252a (200 nM), Tamoxifen (100 nM) or G15 (1 μM), respectively. The number of cells with neurites longer than four times the cell body (α-tubulin staining) or the number of ciliated cells (acetylated α-tubulin staining) was calculated by counting the stained cells in ten fields per slide of three different experiments performed in triplicate.

### Flow Cytometry

As previously described (Sarchielli et al., 2017), after fixation and permeabilization, cells were resuspended in PBS with 1% FBS and incubated with the following primary antibodies: anti-O4 mAb (1:100), anti-MAP2 pAb (1:100), anti-ChAT pAb (1:100) from Millipore; anti-GFAP mAb (1:100), anti-TrkA pAb (1:100) from Santa Cruz Biotechnology, followed by incubation with Alexa Fluor 488 goat anti-mouse IgM (1:200), Alexa Fluor 568 goat anti-rabbit or Alexa Fluor 488 goat anti-mouse IgG (H + L; 1:200) secondary antibodies from Molecular Probes, as appropriate. Cells were analyzed on a FACSCanto II instrument (BD Pharmingen, San Diego, CA, USA) using BD FACSDiva (BD) and FlowJo v10 (Tree Star, Inc., Ashland, OR, USA) softwares.

### Acetylcholine Release Assay

Cells were cultured in choline chloride free medium (Sigma-Aldrich Corp., St. Louis, MO, USA) until subconfluence, then the Ach release in the culture medium was quantified by Choline/Acetylcholine Assay Kit (Abcam), according to the manufacturer’s instructions and using a FlexStation 3 Microplate Reader (Molecular Devices, Sunnyvale, CA, USA).

### Western Blot Analysis

Cells were cultured in serum/phenol red-free condition and treated with NGF (10–100 ng/ml), E2 (10^−10^–10^−7^ M) or G1 (100 nM) in presence or absence of the receptor inhibitors K252a (200 nM), Tamoxifen (100 nM) or G15 (1 μM). Protein extracts (20 μg) were subjected to immunoblotting as previously described (Sarchielli et al., 2014) using the following primary antibodies: anti-p-TrkA pAb (1:1000), anti-pERK 1/2 mAb (1:1000) from Cell Signaling Technologies (Danvers, MA, USA); anti-p-CREB pAb (1:1000), anti-α-tubulin mAb (1:2000), anti-β actin mAb (1:10000), anti-GAP43 mAb (1:1000), anti-STAT1 pAb (1:1000) from Santa Cruz Biotechnology; anti-c fos pAb (1:1000, Sigma-Aldrich); anti-ChAT pAb (1:2000, Millipore).

### Electrophysiology

Whole-cell patch-clamp recordings were performed in −60 mV clamped-cells by using the following solutions: extracellular (mM): HEPES 10, D-glucose 5, NaCl 140, KCl 3, MgCl_2_ 2, and CaCl_2_ 2 (pH 7.4). Pipette (mM): K-Aspartate 130, MgCl_2_ 2, Na_2_-ATP 5, Na_2_-GTP 0.1, EGTA 11, HEPES 10 (pH 7.2). Data were acquired with an Axopatch 200B amplifier (Axon Instruments, Union City, CA, USA), low-pass filtered at 10 kHz, stored and analyzed with a pClamp 9.2 software (Axon Instruments). Detailed protocols used to evoke voltage-dependent K^+^ or Na^+^ currents are described in Supplementary Material. Drugs used were applied by superfusion with a three-way perfusion valve controller (Harvard Apparatus). Current-clamp recordings were performed by applying 12 steps of current injection (300 ms duration; 100 pA increment, from −100 pA to 1000 pA) from the resting membrane potential of the investigated cell, as already described (Coppi et al., 2012) and detailed in Supplementary Material.

### Radioligand Binding Assays

Cell membrane preparations (0.5–0.7 mg/ml) were used for saturation binding assay with [^3^H]NMS (0.05–1.6 nM; Perkin-Elmer Life and Analytical Science), or competition binding assay (0.2 nM) with the muscarinic antagonist metoctramine (0.1 nM–0.1 mM; Sigma-Aldrich) as previously described (Matucci et al., 2016). Nonspecific binding was defined using 10 μM Atropine (Sigma-Aldrich).

### Cell Proliferation Assay

Cell proliferation was determined by MTT assay (Sigma-Aldrich Corp., St. Louis, MO, USA) as previously described (Ambrosini et al., 2015). Cell viability was expressed as relative percentage of viable cells over control, taken as 100% (mean ± SEM) from three separate experiments performed in quadruplicate.

### *In Vivo* Study

#### Animals

All animal procedures were carried out according to the EC Directive 86/609/EEC for animal experiments and National guidelines for animal care with the approval of Italian Ministry of Health (Permit Number: 567/2015-PR). Three-month-old, 230–250 g male Wistar rats (Harlan, Milan, Italy) were used. Either saline (0.9%) or quisqualic acid (QA; 0.12 M) were injected into the right NBM at the following stereotactic coordinates: *AP* = −0.2; *L* = −2.8 and *H* = 6.8 from Bregma (Paxinos and Watson, 2006) in anesthetized rats. The animals were equally divided into four groups (*n* = 5–6 per group): Group I, QA-injected and subjected to intravenous administration of human fetal NBM cells (hfNBMs; 1.5 × 10^6^ in 300 μl PBS) by the tail vein; Group II, QA**-**injected; Group III, saline-injected and subjected to intravenous administration of hfNBMs (1.5 × 10^6^ in 300 μl PBS); Group IV, un-injected rats (controls). One day prior the intravenous administration of cells and for all the length of the experiment rats were treated with Cyclosporine (2 mg/kg/day). Before administration, the cells were labeled with the PKH26 Red Fluorescent dye (Sigma-Aldrich Corp., St. Louis, MO, USA) according to the manufacturer’s instructions. Rats from group I were sacrificed on day 1, 7 and 21 after hfNBMs administration. Rats from the other groups were sacrificed on day 21. Anesthetized (chloral hydrate, 400 mg/kg i.p.) rats were perfused transcardially with 0.9% saline followed by 4% paraformaldehyde and brains were paraffin embedded. Livers from rats subjected to intravenous administration of hfNBMs were harvested and analyzed to detect the presence of PKH26 labeled cells in systemic organs.

#### Immunohistochemistry

Immunohistochemical analyses of rat brains were performed on 5.0 μm coronal paraffin-embedded sections. Anti-GFAP (1:1000; Agilent Technologies, Santa Clara, CA, USA) and anti-ChAT (1:200; Millipore) pAbs were used to detect astrocytes and cholinergic neurons, respectively. ChAT-positive cells in the NBM were counted under a 10× objective. Five sections per animal, anteroposterior standardized with respect to the injection site and spaced 50–100 μm from one another, were analyzed. The total number of ChAT-positive cells in the QA-injected NBM was averaged, expressed as a percentage of that counted in the saline-injected NBM (*n* = 3 per group), and analyzed using Prism 5.0 (GraphPad Software, San Diego, CA, USA).

#### Morris Water Maze Test

During the third week after hfNBMs administration rats were tested in the Morris Water Maze (MWM). The MWM apparatus consisted of a circular pool (1.6 m in diameter and 0.36 m high) made of green plastic. The pool was filled to a depth of 20 cm with water (24–25°C) that was made dark by the addition of non-toxic dark paint. Rats were tested in the reference memory version of MWM with the procedure previously described for mice (Grossi et al., 2013).

#### Step-Down Inhibitory Avoidance Task

The day after the end of MWM task the animals were tested in the Step-Down inhibitory avoidance task as previously described for mouse (Grossi et al., 2009) with some modifications. The apparatus was an open field plexiglas box (50 × 25 × 25 cm) with a steel rod floor and a plexiglas platform (5 × 8 × 25 cm) set on the grid floor to which intermittent electric shocks were delivered.

### Statistics

Data are expressed as mean ± SEM. Student’s paired or unpaired *t*-tests or One- and two-way analysis of variance (ANOVA) followed by Newman–Keuls or Bonferroni post-test analysis were performed, as appropriate, in order to determine statistical significance (set at *p* < 0.05). Data were analyzed using software package GraphPad Prism (GraphPad Software).

## Results

### Phenotypic Characterization of hfNBMs

**Figure 1:**
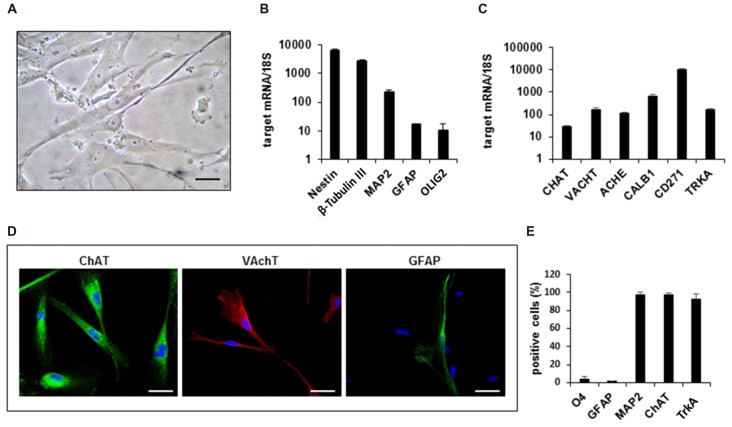
Gene expression profile and phenotypic characterization of hfNBMs. **(A)** Phase contrast microphotographs showing cell morphology at passage 4 (scale bar 50 μm). **(B,C)** Relative mRNA expression by quantitative real time-PCR (qRT-PCR) analysis of target genes normalized over 18S ribosomal RNA subunit, taken as the reference gene; data are mean ± SEM (*n* = 6). **(D)** Representative images of cells expressing ChAT, VAchT and GFAP (DAPI counterstained nuclei, scale bar 50 μm). **(E)** Flow cytometric analysis for O4, GFAP, MAP2, ChAT e TrkA proteins; the bar graph shows the percentage of positive cells reported as mean ± SEM (*n* = 4).

In order to isolate human cholinergic neurons from NBM, we dissected and dissociated the corresponding region within the BF of 12-week old human fetuses. The cell suspensions obtained were plated and maintained in Coon’s medium supplemented with fetal bovine serum. One week after plating, the cells started to emerge from aggregates and grew as a monolayer population of adherent cells. At this stage, the cells were harvested and replated into fresh medium generating a primary cell culture of hfNBMs neurons (Figure 1A) that was propagated and characterized at different passages.

In order to define the identity of the hfNBM population, we analyzed the expression of immature and mature neural lineage cell markers by qRT-PCR, immunofluorescence analysis and flow cytometry. As shown in Figure 1B, hfNBMs expressed high abundance of both nestin and β-tubulin III transcripts, which are indicative of neural precursors committed toward the neuronal lineage. Accordingly, we found that the mRNA expression of MAP2, a neuronal marker, was more abundant than the markers of either astroglial (glial fibrillary acidic protein, GFAP) or oligodendroglial (OLIG2) lineages. In addition, hfNBMs expressed a panel of specific genes for NBM cholinergic identity, such as those codifying for enzymes essential for Ach synthesis (choline acetyltransferase, CHAT) and degradation (ACHE), the Ach vescicular transporter (VACHT), the calcium binding protein calbindin 1 (CALB1), and both types of the NGF receptors (TRKA, CD271; Figure 1C). This gene expression profile was retained by cells throughout the different passages in culture (p. 4–p.26).

Immunocytochemical analysis performed in hfNBMs confirmed the prominent neuronal phenotype with a strong immunopositivity to ChAT and VAchT protein expression and a rare detection of GFAP-positive cells (Figure 1D). To provide a quantitative characterization of the hfNBMs phenotype, we used flow cytometry. As shown in Figure 1E, the majority of cells was positive for MAP2 (97.25 ± 2.75%), ChAT (97.03 ± 2.09%) and TrkA (92.45 ± 5.65%), with a very low percentage of cells being positive for glial markers, such as GFAP (1.65 ± 0.05%) and O4 (4.05 ± 2.55%). Finally, the cholinergic phenotype of hfNBM cells was confirmed by the detection of basal release of Ach (1.23 ± 0.76 nmol/ml) in the culture medium.

### Functional Characterization of hfNBM Cells

**Figure 2:**
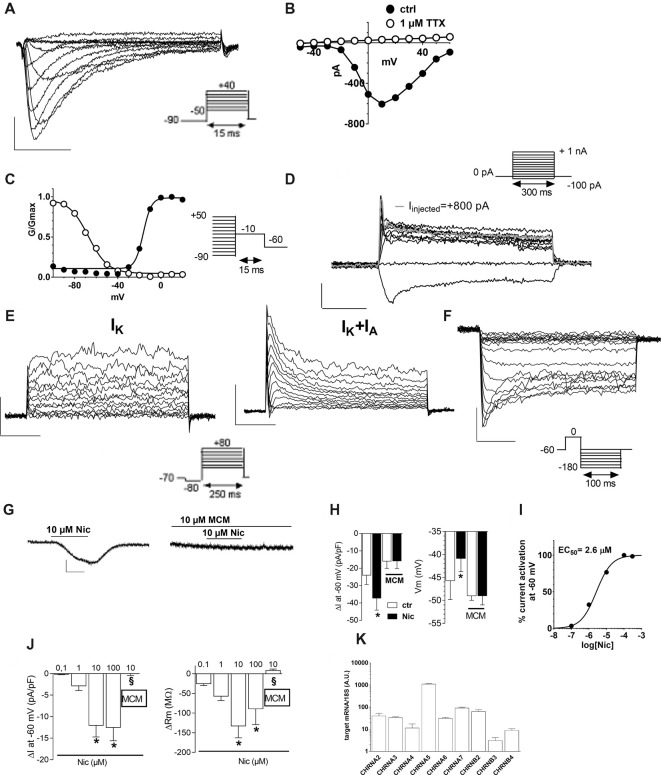
Electrophysiological characterization of hfNBMs. **(A)** Original Na^+^ currents (I_Na_) recorded by whole-cell patch clamp technique in a representative cell (at p19). Calibration: 200 pA, 5 ms. **(B)** Current to voltage relationship (I–V plot) of I_Na_ recorded in the same cell in absence (●) or in presence (○) of 1 μM tetrodotoxin (TTX). **(C)** Activation (●) and inactivation (○) curves of I_Na_ evoked in the same cell. Black lines represent fittings with Boltzmann equations (V_1/2_ activation = −14.73 ± 1.44 mV; slope = 4.12 ± 0.38; V_1/2_ inactivation = −56.25 ± 3.60 mV; slope = 5.85 ± 4.10). **(D)** Original whole-cell current-clamp recordings in a cell at p19 showing a resting membrane potential of −48 mV. A prominent sag is observed at −100 pA of current injection. Note that +800 pA current injection elicited a single, small amplitude, action potential (gray trace). No repetitive firing activity was elicited at higher values of current injection (+900 pA; +1 nA). Scal bars: 100 ms; 20 mV. **(E)** Original current traces elicited by a depolarizing voltage step protocol in a representative cell (at p16) expressing only delayed rectifier (I_K_) currents (left panel) or in a different cell (at p26) expressing either I_K_ or transient I_A_ currents (right panel). Calibration: 500 pA, 50 ms. **(F)** Original K_ir_ current traces elicited by a hyperpolarizing voltage step protocol in a representative cell (at p17). Calibration: 200 pA, 25 ms. **(G,H)** Original current traces **(G)** and pooled data (**H**, left panel) recorded in −60 mV clamped cell where nicotine (Nic, 10 μM) activated an inward current blocked by the selective antagonist Mecamilamine (MCM, 10 μM). Scale bars: 100 pA, 1 min. Right panel: pooled data indicating that, concomitantly with inward current activation, Nic also concentration-dependently depolarized Vm (*p < 0.05, *n* = 12). **(I)** Concentration-response curve of Nic (EC_50_ = 2.6 μM, C.L. 2.05–3.4 μM) in hfNBMs. **(J)** Pooled data indicating the concentration-dependent effect of Nic, alone or in combination (10 μM Nic) with MCM, on inward current and Rm in hfNBMs. *p < 0.05 vs. 0.1 μM Nic; ^§^*p* < 0.05 vs. 10 μM Nic. Data are expressed as mean ± SEM from at least *n* = 4. **(K)** Relative mRNA expression by qRT-PCR analysis of nicotinic receptor subunits normalized over 18S ribosomal RNA subunit, taken as the reference gene, and reported as mean ± SEM (*n* = 6).

We next profiled the functional features of hfNBMs by studying their electrophysiological properties. The present results have been obtained by patch-clamp whole cell recordings from 102 cells (from p14 to p26). The registered electrophysiological parameters, including resting membrane potential (Vm), membrane resistance (Rm) and membrane capacitance (Cm), showed values typically found in neurons, as reported in Table 1. More relevantly, 89% of hfNBMs tested (91 out of 102) exhibited fast inward currents in response to a step depolarization (Figure 2A), indicating the expression of functional voltage-gated Na^+^ channels (Table 1). These currents were rapidly activating and inactivating, tetrodotoxin (TTX)-sensitive, and presented an I–V plot and an activation-inactivation curve (activation curve: V_1/2_ = −14.7 ± 1.4 mV; slope factor = 4.2 ± 0.4; inactivation curve: V_1/2_ = −21.7 ± 6.2; slope factor = 3.7 ± 0.2) typical of Na^+^ currents (I_Na_) recorded in neurons (Figures 2B,C), thus providing functional validation of the neuronal profile of the hfNBMs.

**Table 1:** Electrophysiological properties of hfNBMs.

Membrane potential (Vm; mV)	−44.8 ± 3.2 (*n* = 102)
Membrane resistance (Rm; MΩ)	749.7 ± 77.7 (*n* = 102)
Membrane capacitance (Cm; pF)	30.1 ± 2.1 (*n* = 102)
Sodium current (I_Na_) amplitude at 0 mV (pA)	−993.8 ± 72.4 (*n* = 91)
K^+^ current (I_K_) amplitude at +80 mV (pA)	556.4 ± 50.7 (*n* = 102)

*Data are presented as mean ± SEM of n cells*.

In order to elucidate hfNBM firing activity cells presenting I_Na_ currents were investigated under current-clamp configuration. A prominent sag was observed upon negative current injection (−100 pA, Figure 2D) in the majority of cells investigated (see Table 2). A single, small amplitude, action potential (AP) was elicited in some cells upon depolarizing current injections (from +500 pA). A modest fast AHP (fAHP) followed the single AP. Repetitive firing was never observed, nor slow AHP (sAHP) at the end of the current step. Quantitative analysis of current-clamp parameters is reported in Table 2.

**Table 2:** Quantitative analysis of current-clamp parameters recorded in hfNBMs.

Passage in culture *(p)*	*n*. cells with AP	*n*. cells with SAG	Sag ratio	AP threshold (mV)	AP amplitude (mV)	AP half width (ms)	fAHP (mV)
p15 (*n* = 12)	*n* = 4	*n* = 10	2.9 ± 0.2	−32.2 ± 1.6	21.8 ± 2.0	1.95 ± 0.1	8.01 ± 3.3
p19 (*n* = 13)	*n* = 3	*n* = 13	3.1 ± 0.4	−41.1 ± 2.1	25.5 ± 4.6	1.06 ± 0.3	9.3 ± 3.3

*Data are presented as mean ± SEM of n cells*.

Concerning investigation of K^+^ currents, we applied a voltage step protocol (Figure 2E, inset) able to activate both slowly activating and not inactivating (delayed rectifier) K^+^ currents (I_K_: Figure 2E, left panel) or fast activating and rapidly inactivating K^+^ currents (I_A_: Figure 2E, right panel), in accordance to Sah (1995). Contrarily to I_A_ currents, detected only in a minority of hfNBMs (37.2%; 38 out of 102), I_K_ currents were observed in all cells tested. We also applied a specific voltage protocol (Figure 2F, inset) to activate inwardly rectifying K^+^ currents (Kir), which were recorded in about 40% of cells (43 out of 102; Figure 2F). All these currents (I_K_, I_A_ and Kir) were abolished when extra- and intracellular K^+^ ions were replaced by equimolar Cs^+^ (not shown), thus confirming that they are potassium currents.

We further examined the presence of functional cholinergic (nicotinic and muscarinic) receptors in hfNBMs. First, we investigated the expression of potential nicotinic receptors by applying the selective agonist nicotine (Nic). When a single hfNBM cell was voltage clamped at −60 mV, Nic (10 μM, 2 min application) evoked an inward current (Figure 2G, left panel) completely inhibited by the selective Nic antagonist mecamilamine (MCM, 10 μM, Figure 2G, right panel and Figure 2H, left panel; *p* < 0.05). The effect of Nic was observed in 76.3% of tested cells (29 out of 38 cells) and was concentration-dependent (Figure 2J, left panel) with EC_50_ = 2.6 μM (confidential limits: 2.05–3.4 μM; Figure 2I). Concomitantly with inward current activation, Nic also induced a MCM-sensitive depolarization of Vm (Figure 2H, right panel) and a concentration-dependent decrease in Rm (Figure 2J, right panel).

**Figure 3:**
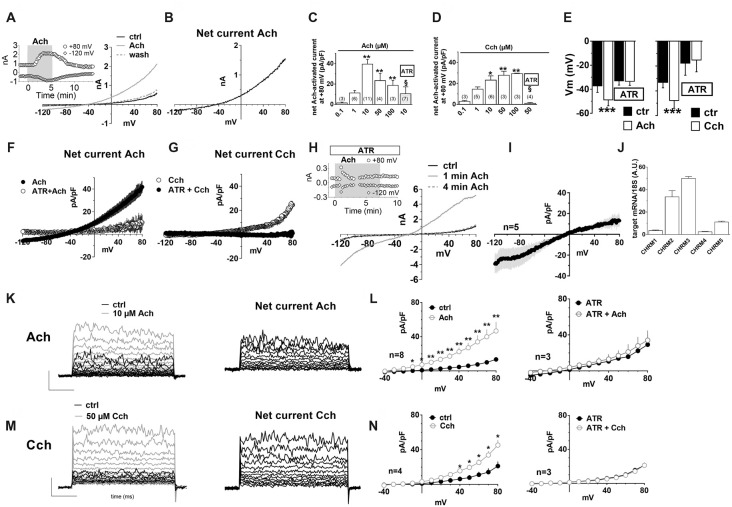
Muscarinic receptor activation by acetylcholine (Ach) or carbachol increases outward K^+^ currents in hfNBMs. **(A)** Original patch clamp current traces recorded in a representative cell where a voltage ramp protocol (−120/+80 mV, 800 ms) was applied before (ctrl), during and after (wash) 10 μM Ach application. Inset: time course of ramp-evoked currents at +80 mV (open circles) or at −120 mV (open diamonds) in the same cell. **(B)** Net Ach-sensitive current, obtained by subtraction of the control ramp from that recorded in Ach, in the same cell. **(C–E)** Pooled data of Ach **(C)** and Carbachol (Cch, **D**) concentration-dependent effects on ramp current measured at +80 mV and on membrane potential (Vm, **E**). All these effects induced by muscarinic agonists are blocked by 100 nM atropine (ATR) **(C,D)** *p < 0.05; **p < 0.01, ***p < 0.001 vs. 0.1 μM Ach; ^§^*p* < 0.05 vs. 10 μM Ach, One-way analysis of variance (ANOVA), Newman-Keuls post test. Number of cells tested is reported in parenthesis. **(E)** ***p < 0.001 vs. respective ctrl, paired Student’s *t*-test, *n* = 12 Ach alone; *n* = 8 Ach + ATR; *n* = 6 Cch alone; *n* = 4 Cch + ATR. **(F)** Averaged Ach-sensitive currents recorded in the absence (filled circles; *n* = 12) or in the presence (open circles; *n* = 10) of 100 nM ATR. **(G)** Averaged Cch-sensitive currents recorded in the absence (filled circles; *n* = 6) or in the presence (open circles; *n* = 5) of 100 nM ATR. **(H)** Original current traces evoked by a voltage ramp protocol in a representative cell in the presence of ATR immediately before (black trace: ctrl) or during (1 min: gray trace; 4 min: dotted gray trace) 10 μM Ach application. Inset: Time course of ramp-evoked current at +80 mV (open circles) or −120 mV (open diamonds) in the same cell. Note the “nicotinic-like” effect of Ach revealed in the presence of ATR. **(I)** Averaged net Ach-sensitive current, obtained by subtraction of the control ramp from the Ach trace, recorded in the presence of 100 nM ATR in five cells investigated. **(J)** Relative mRNA expression by qRT-PCR analysis of muscarinic receptor subunits normalized over 18S ribosomal RNA subunit, taken as the reference gene, and reported as mean ± SEM (*n* = 6). **(K)** Left panel: original current traces evoked by a voltage step protocol (from −40 to +80 mV, 10 mV steps, 200 ms duration, pre-step = −80 mV) in a representative hfNBM cell before (black trace) or during (gray trace) Ach (10 μM) application. Right panel: net Ach-activated current, obtained by subtraction of the control ramp from the Ach trace, in the same cell. **(L)** Averaged I–V plots of step-evoked currents recorded in Ach alone (10 μM, *n* = 8, left panel) or in the presence of ATR (100 nM, *n* = 3, right panel). *p < 0.05; **p < 0.01 vs. ctrl; paired Student’s *t*-test. **(M)** Left panel: original current traces evoked by a voltage step protocol in a representative cell before (black trace) or during (gray trace) Cch (50 μM) application. Right panel: net Ach-activated current, obtained by subtraction of the control ramp from the Cch trace, in the same cell. **(N)** Averaged I–V plots of step-evoked currents recorded in Cch alone (50 μM, *n* = 4, left panel) or in the presence of ATR (100 nM, *n* = 3, right panel). *p < 0.05; **p < 0.01 vs. ctrl.

To explore the functional effects of muscarinic receptors, we applied Ach or carbachol (Cch). A depolarizing voltage-ramp protocol was used to elicit a wide range of overall voltage-dependent currents in hfNBMs before, during and after Ach or Cch application. Depolarizing voltage ramps in hfNBMs evoked a majority of K^+^ currents plus a mixture of other voltage-gated currents as indicated by their reversal potential of about −45 mV (Figure 3A: black trace), which does not coincide with the predicted K^+^ equilibrium potential in our experimental conditions (E_K_ = −95.2 mV). However, the most of ramp currents are K^+^ currents since they are almost absent in Cs^+^-replacement experiments (Supplementary Figure S1A). Figure 3A shows a typical experiment where Ach (10 μM) reversibly increased ramp-evoked K^+^ currents in a single cell. The Ach-sensitive current is an outward current with an activation voltage around −40 mV (Figure 3B). Ach-induced current increase was concentration-dependent (Figure 3C; *p* < 0.01), prevented by Cs^+^ replacement (Supplementary Figure S1B) and blocked by the muscarinic antagonist atropine (ATR, 100 nM, Figure 3C; *p* < 0.05). The maximal effect of Ach was elicited at a concentration of 10 μM with a “bell-shaped” curve observed at higher concentrations, probably due to higher rate of Ach degradation by AchE, which is expressed by hfNBMs. Cch mimicked Ach effects on ramp-evoked currents as it concentration-dependently increased outward K^+^ currents (Figure 3D). Finally, either Ach or Cch induced an ATR-sensitive Vm hyperpolarization (Figure 3E; *p* < 0.001) in line with K^+^ channel opening. Figures 3F,G show Ach- and Cch-sensitive currents in the absence or presence of ATR, respectively. When tested on the voltage-step protocol to specifically activate K^+^ currents, either Ach (Figure 3K) or Cch (Figure 3M) increased currents with kinetic properties and I-V plot typical of delayed rectifier I_K_ conductances (Figures 3K,L, left panels; Figures 3M,N, left panel); both effects were blocked by ATR (Figures 3L,N, right panels). Thus, we conclude that muscarinic receptor activation in hfNBMs by Ach or Cch enhances delayed rectifier I_K_ currents.

It should be mentioned that, when Ach was applied in the presence of ATR, we observed a transient increase in the inward component of ramp-evoked currents (Figure 3H). This effect, observed only in a subset of hfNBMs (4 out of 7), peaked within the first minute of application and rapidly disappeared before agonist washout (Figure 3H, inset). The reversal potential of Ach-sensitive current recorded in the presence of ATR was around 0 mV (Figure 3I), coherently with the activation of rapidly desensitizing, non-selective cation channels, such as nicotinic receptors.

In order to define which types of cholinergic receptors were expressed by hfNBMs, we analyzed the mRNA expression of the different neuronal subunits of nicotinic receptors (from α2 to α7 and from β2 to β4) along with the different subtypes of muscarinic receptors (from M1 to M5). As shown in Figure 2K, hfNBMs expressed all the nicotinic receptor subunits analyzed, even though at different levels, with α5 subunit (CHRNA5) being the most abundant and β3 subunit (CHRNB3) the less expressed (Figure 2K). Similarly, the expression of all the muscarinic receptor isoforms was detected (Figure 3J). In particular, we found high levels of the M2 (CHRM2) and M3 (CHRM3) subtypes. It is known that the M2 isoform is particularly important for cholinergic neuron function as it acts as a pre-synaptic autoreceptor mediating an inhibitory action on Ach release. Hence, the presence of muscarinic receptors, and in particular M2 sites, in hfNBMs was further investigated by radioligand binding analysis. Saturation curves obtained at equilibrium conditions with the non-selective muscarinic antagonist [^3^H]-NMS resulted in a maximum binding capacity (Bmax) of 83.52 ± 18.72 fmol/mg protein and in a dissociation constant (K_D_) of 0.07 ± 0.01 nM, thus indicating the presence of high affinity muscarinic cholinergic receptor binding sites (Supplementary Figure S2A). To detect the presence of M2 sites we performed competition binding experiments, using methoctramine, a muscarinic receptor antagonists that binds to M2 with high affinity. [^3^H]-NMS binding was completely inhibited by methoctramine providing evidence that M2 is expressed in these cells (Supplementary Figure S2B). The competition-binding assays performed with methoctramine yielded displacement curves, best described with a two-site binding model (pK_iH_ = 8.18 ± 0.37; pK_iL_ = 6.21 ± 0.21; *n* = 3; *p* < 0.01; Supplementary Figure S2B).

### NGF Activates a Functional TrkA Signaling Pathway in hfNBMs

**Figure 4:**
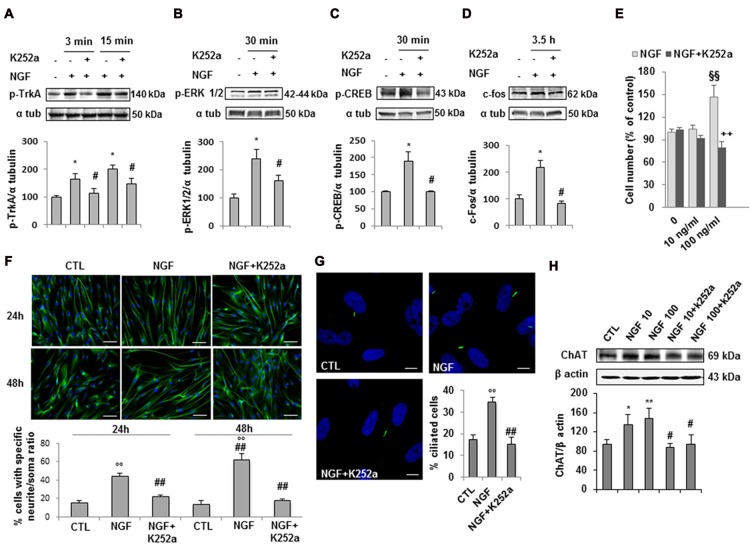
NGF/TrkA signaling activation and effects of NGF on hfNBMs. **(A–D)** Immunoblotting of TrkA, ERK 1/2 and CREB phosphorylation and c-fos expression induced by 100 ng/ml NGF for 3, 15, 30 min (min) or 3.5 h in presence or absence of the receptor inhibitor K252a (200 nM); band intensity was normalized over α-tubulin signal and expressed as % of untreated cells; data are mean ± SEM (*p < 0.05 vs. untreated cells, ^#^*p* < 0.05 vs. NGF, *n* = 3). **(E)** MTT assay of hfNBMs untreated or treated with 10–100 ng/ml NGF for 24 h; data are expressed as percentage of untreated cells and are mean ± SEM (^§§^*p* < 0.005 vs. untreated cells, ^++^*p* < 0.005 vs. NGF, *n* = 3). **(F)** Neurite outgrowth analysis by α-tubulin staining of hfNBMs treated or not with 100 ng/ml NGF for 24 or 48 h in presence or absence of K252a (200 nM); the graph shows the percentage of cells with neurites longer than four times the cell body, calculated by counting 10 fields per slide of three separate experiments (^○○^*p* < 0.001 vs. CTL, ^##^*p* < 0.001 vs. NGF; DAPI counterstained nuclei, scale bar 100 μm). **(G)** Acetylated α-tubulin staining of primary cilium (green) in hfNBMs (DAPI counterstained nuclei, scale bar 10 μm). The number of ciliated cells was counted in 10 different random fields and expressed as percentage of DAPI-stained total cells; data are mean ± SEM of three separate experiments (^○○^*p* < 0.001 vs. CTL, ^##^*p* < 0.001 vs. NGF). **(H)** Immunoblotting of ChAT expression in cells treated or not with 10–100 ng/ml NGF for 24 h in presence or absence of 200 nM K252a; band intensity was normalized over β actin signal and expressed as % of control; data are mean ± SEM (*p < 0.05, **p < 0.01 vs. CTL; ^#^*p* < 0.05 vs. respective NGF; *n* = 3).

Experimental evidence indicates that NGF/TrkA signaling supports survival, maintenance, connectivity and function of the brain cholinergic neurons. As we demonstrated that hfNBMs express the TrkA receptor (Figures 1C,E), we next questioned whether the formation of a NGF-TrkA complex activated a functional TrkA signaling pathway and assessed the phosphorylation of TrkA (pTrkA) and the activation of TrkA down-stream effectors (p-ERK1/2, p-CREB or c-fos). After 24 h of serum starvation, cells were stimulated with 100 ng/ml NGF and then harvested at different time points. Western blot analysis with specific antibodies revealed that NGF significantly induced pTrkA within 3′-15′ and p-ERK1/2 or p-CREB at 30’, whereas maximum activation of c-fos was within 3.5 h (Figures 4A–D; *p* < 0.05). Preincubation with the specific receptor inhibitor K252a prevented all these effects. To test the role of NGF on hfNBMs proliferation, we treated cells with two different concentrations of NGF (10 and 100 ng/ml) for 24 h. Using the colorimetric MTT assay we found the 100 ng/ml NGF caused a significant increase in cell number (*p* < 0.005 vs. untreated cells), which was blocked by the addition of K252a (Figure 4E; *p* < 0.005 vs. NGF). The effect of NGF on cholinergic neuronal differentiation was determined by analyzing neurite outgrowth. Serum-starved cells were incubated with 100 ng/ml NGF for 24 and 48 h, and the occurrence of neurite elongation was evaluated by immunofluorescent detection of α-tubulin, in comparison with untreated cells (Figure 4F). Neurite outgrowth was determined by measuring the ratio between the longest neurite length and the cell body diameter and calculating the percentage of cells with a particular ratio, as already described (González-Martínez et al., 2004; Sarchielli et al., 2014). As shown in Figure 4F, in the absence of treatments, about 15% of cells (15.6 ± 2% at 24 h and 14.3 ± 3.4% at 48 h) showed neurites longer than four times the cell body, while the percentage was significantly increased after 100 ng/ml NGF treatment at both 24 h (44.5 ± 3.2%, *p* < 0.001) and 48 h (62.8 ± 6.5%, *p* < 0.001), an effect prevented by preincubating cells with K252a at both time points. The effect of NGF on neuritogenesis was confirmed by analyzing growth associated protein 43 (GAP43) protein expression (Supplementary Figure S3). Interestingly, acetylated α-tubulin immunostaining, which specifically detects the formation of a primary cilium, revealed that NGF treatment significantly increased the percentage of cells exhibiting this organelle (34.8 ± 1.8%; Figure 4G), as compared to untreated cells (17.3 ± 2.3%; *p* < 0.0001), an effect inhibited by K252a (15.1 ± 3.2%). Because NGF is an essential regulator of cholinergic neuronal phenotype, we also investigated whether the neurotrophin could modulate ChAT expression. As shown in Figure 4H, either 10 or 100 ng/ml NGF significantly increased ChAT protein expression, compared with untreated cells (*p* < 0.05 and *p* < 0.01, respectively), an effect blocked by K252a.

### Effects of Estrogen on hfNBMs

**Figure 5:**
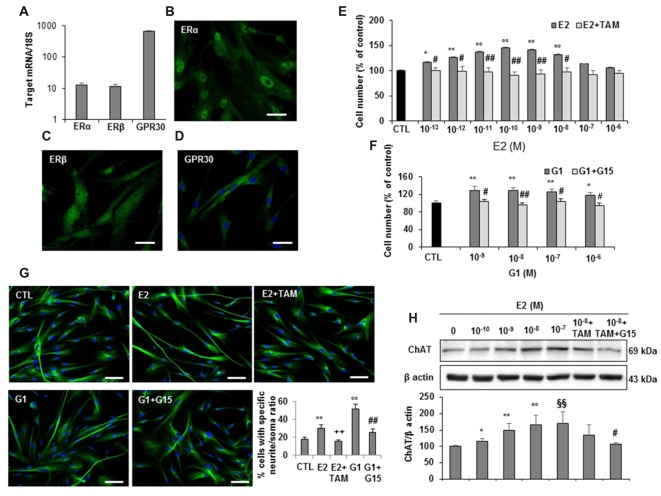
Estrogen receptors expression and estrogen effects on hfNBMs. **(A)** Relative mRNA expression by qRT-PCR analysis of ERα, ERβ and GPR30 receptors normalized over 18S ribosomal RNA subunit, taken as the reference gene, and reported as mean ± SEM (*n* = 6). **(B–D)** Immunocytochemical detection of ERα, ERβ and GPR30 in hfNBMs (DAPI counterstained nuclei in **(D)**, scale bar 50 μm). **(E,F)** MTT analysis of hfNBMs treated or not (CTL) with increasing concentrations of E2 (10^−13^–10^−6^ M) or the GPR30 agonist G1 (10^−9^–10^−6^ M) for 24 h in the presence or absence of the ERα/ERβ antagonist tamoxifen (TAM; 100 nM) or the GPR30 antagonist G15 (1μM), respectively (*p < 0.05, **p < 0.01, ^○○^*p* < 0.001 vs. CTL; ^#^*p* < 0.05, ^##^*p* < 0.001 vs. treated cells; *n* = 3). **(G)** Neurite outgrowth analysis by α-tubulin staining of hfNBMs treated with 10 nM E2 for 24 h in presence or absence of TAM (100 nM) or treated with 100 nM G1 for 24 h in presence or absence of G15 (1 μM); the graph shows the percentage of cells with neurites longer than four times the cell body calculated by counting ten fields per slide of three separate experiments (**p < 0.01, ^○○^*p* < 0.001 vs. CTL; ^##^*p* < 0.001, ^++^*p* < 0.005 vs. treated cells; DAPI counterstained nuclei, scale bar 100 μm). **(H)** Immunoblotting of ChAT expression in serum-starved cells treated or not (CTL) with increasing concentrations of E2 (0.1–100 nM) or with 1 nM E2 in the presence of TAM (100 nM) alone or in combination with G15 (1 μM) for 24 h; band intensity was normalized over β actin signal and expressed in % of CTL; data are mean ± SEM (*p < 0.05, **p < 0.01, ^○○^*p* < 0.001, ^§§^*p* < 0.005 vs. CTL; ^#^*p* < 0.05 vs. E2 10 nM, *n* = 3).

Based on the evidence in literature about the possible association between estrogen plasma levels and AD incidence, we next investigated whether hfNBMs expressed estrogen receptors and responded to 17β-estradiol (E2). As shown in Figure 5, mRNA expression analysis (Figure 5A) and immunofluorescent detection (Figures 5B–D) demonstrated that hfNBMs possessed both classical estrogen receptors (ERα and ERβ), along with the transmembrane G protein-coupled ER (GPR30/GPER1), which is known to mediate rapid non-genomic actions. Increasing concentrations of E2 (0.1–100 nM) or G1 (1–1000 nM), a specific GPR30 agonist, determined a dose-dependent increase in cell number after 24 h exposure, which was reverted by the pre-treatment with either the ERα/ERβ antagonist tamoxifen (100 nM) or the selective GPR30 antagonist G15 (1 μM), respectively (Figures 5E,F). Similar effects were observed in terms of hfNBMs differentiation, since 24 h exposure to 10 nM E2 or 100 nM G1 significantly induced neuritogenesis, an effect fully abolished by the respective antagonists tamoxifen or G15 (Figure 5G; *p* < 0.05). In addition, E2 (0.1–100 nM, 24 h) determined a significant dose-dependent increase in ChAT protein expression (*p* < 0.05 for each E2 concentration vs. control), which tended to be reduced by tamoxifen pre-treatment, although without reaching statistical significance (Figure 5H). Pretreating cells with both G15 and tamoxifen significantly counteracted E2 effect (Figure 5H), thus suggesting a crucial involvement of GPR30 activation in inducing estrogen-mediated ChAT expression. Accordingly, G1 fully mimicked E2 effects on ChAT expression (Supplementary Figure S4).

### hfNBMs Improve Memory Functions in NBM-Lesioned Rats

**Figure 6:**
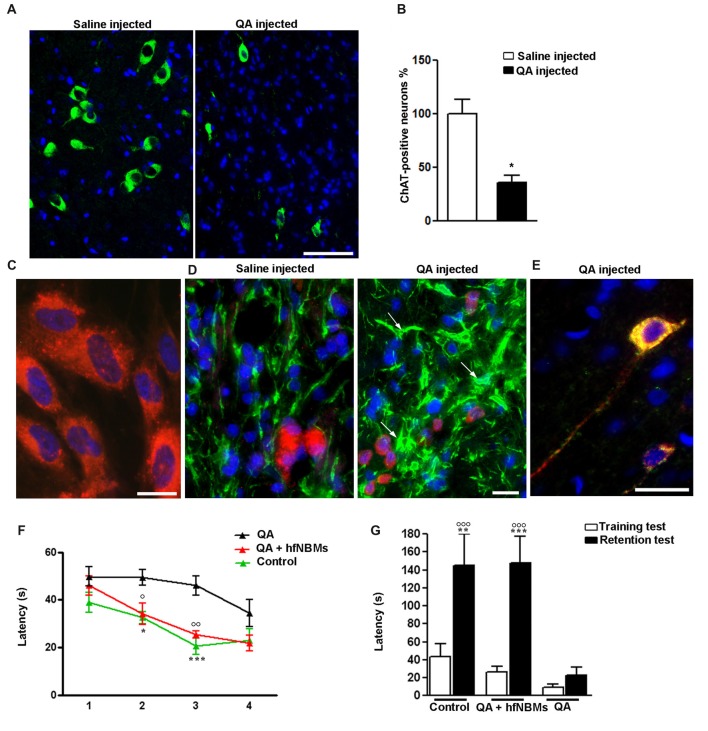
Effects of hfNBMs intravenous administration in nucleus basalis of Meynert (NBM)-lesioned rats. **(A,B)** Immunohistochemical detection **(A)** and quantification **(B)** of ChAT positive cells in the right NBM of quisqualic acid (QA)-injected rats, compared to saline-injected rats; nuclei are counterstained with DAPI (scale bar 50 μm). Data are expressed as percentage of ChAT-positive neurons (mean ± SEM), taking saline-injected rats as 100% (*p < 0.001; *n* = 3 per group). **(C)** Representative image showing incorporation of the red fluorescent dye PKH26 in hfNBM cells (DAPI counterstaining, scale bar 10 μm). **(D)** Localization of PKH26-labeled hfNBM cells (red) and evaluation of astrogliosis (white arrows) by anti-GFAP staining (green) in the right NBM of QA-injected compared to saline-injected rats (DAPI counterstaining, scale bar 10 μm). **(E)** Merge of dual labeling of ChAT (green) and the dye PKH26 (red) in NBM sections of QA-injected rats at day 21 (scale bar 20 μm); ChAT is expressed in hfNBM cells as demonstrated by the yellow color. **(F,G)** Working memory assessment: **(F)** Morris water maze (MWM) test, QA-injected rats required significantly more time to find the platform at day 2 and 3 (*p < 0.05, ***p < 0.001, respectively) compared to controls, while the QA + hfNBMs group showed a response similar to controls and significantly different from QA rats (^○^*p* < 0.05 day 2 and ^○○^*p* < 0.01 day 3; *n* = 5–6 per group); **(G)** Step-down inhibitory avoidance test, the training test showed no significant differences between groups. The 24 h retention test showed increased latencies in controls (**p < 0.01) and QA+ hfNBMs rats (***p < 0.001) vs. respective training latencies. In QA rats retention latencies were significantly reduced respect to controls and QA+ hfNBMs (^○○○^*p* < 0.001) and not significantly different from training latencies (*n* = 5–6 per group).

To test the ability of hfNBMs in improving injured cholinergic function, we performed *in vivo* experiments using an acute animal model of AD, generated by the unilateral injection of quisqualic acid (QA) into the right NBM of adult male rats. In accordance with previous report (Bartolini et al., 1996), 21 days after NBM lesioning the number of magnocellular ChAT-positive neurons in the QA-injected side was significantly reduced (−64%) compared to saline-injected NBM (Figures 6A,B, *p* < 0.001). Intravenous administration of hfNBMs was performed in subgroups of QA- and saline-injected rats. To evaluate whether the intravenously administered cells reached the lesioned NBM, hfNBMs were labeled with the red fluorescent dye PKH26 (Figure 6C). As shown in Figure 6D, the immunohistochemical analysis of astrogliosis revealed the presence of reactive GFAP-positive astrocytes with enlarged cell bodies and long processes in NBM sections from QA- but not saline-injected rats (Figure 6D). The presence of PKH26-labeled hfNBMs was detected both in the QA- and saline-injected NBM (Figure 6D). Immunofluorescent staining with ChAT antibody of NBM sections from the experimental rats showed that PKH26-labeled cells were ChAT-positive, as exemplified by the merge image from QA-injected rats at day 21 (Figure 6E). No PKH26-labeled cells were detected in uninjured brain areas, including cortex, striatum, medial septum and peduncolopontine nuclei of both cerebral hemispheres, nor in the left unlesioned NBM.

The effects of hfNBMs administration on cognitive functions were assessed with both MWM and step down inhibitory avoidance tests. At day 21, rats from each experimental group (*n* = 5–6 per group) were trained for 4 days in the MWM task to learn where the hidden platform was located. The rats were naive to the water maze and showed no deficiencies in swimming abilities, directional swimming towards the platform, or climbing onto a hidden platform during training trials. As shown in Figure 6F, QA-injected rats required significantly more time to find the platform compared to controls (*p* < 0.05, day 2, and *p* < 0.001, day 3). Interestingly, rats from the QA-injected + hfNBMs group were good swimmers and showed an appropriate swim-search response after being placed in water, significantly shortening the escape latency during the 2- and 3-day acquisition phase compared to QA-injected group (Figure 6F; *p* < 0.05, day 2 and *p* < 0.01, day 3). At day 4 of acquisition phase, QA-injected rats shortened the escape latency with no significant differences when compared to QA+ hfNBMs or control rats (Figure 6F). No differences were detected in the escape latency in all acquisition phase sessions between control and saline-injected + hfNBMs rats (data not shown). The same animals were then tested for the step-down inhibitory avoidance (*n* = 5–6 per group; Figure 6G). No significant differences were observed among all groups during the training test. In the 24 h retention test step-down latencies recorded for controls and QA+ hfNBMs rats were significantly increased (*p* < 0.01 and *p* < 0.001 vs. their respective training latencies). In QA rats retention latencies were significantly reduced compared to controls and QA+ hfNBMs (*p* < 0.001) and not significantly different from training latency, indicating that QA rats were unable to memorize the punishment and to perform the inhibitory avoidance. Administration of hfNBMs to QA rats significantly improved their performance, which reached the level displayed by control rats (Figure 6G). No differences were detected during retention test between control and saline injected + hfNBMs rats (data not shown).

## Discussion

The impairment of the BF cholinergic system, especially NBM, is a crucial event in the progression of memory and cognitive decline associated to neurodegenerative disorders, such as AD. It therefore represents the main target for therapeutic approaches (Ferreira-Vieira et al., 2016). The most efficient treatment should be able to direct a regenerative process of the damaged brain areas, a possibility currently under consideration in the attempt to establish efficient stem cell-based replacement therapies for AD. Studies on derivation of functional BF cholinergic neurons from stem cells are limited (Bissonnette et al., 2011; Liu et al., 2013; Yue et al., 2015) and, although the regenerative potential has been proved in AD animal models, the actual application in clinical protocols seems far to be reached. Besides the generation of functionally integrated stem cell-derived cholinergic neurons, major efforts should be directed towards unraveling those mechanisms that may protect NBM neurons from degenerative insults, with the main aim of identifying efficient strategies to prevent AD pathogenesis. In this study, we provide for the first time a comprehensive phenotypic and functional characterization of primary human cholinergic neurons isolated from the fetal NBM. They may represent not only an optimal resource to study developmental processes of neurons already committed towards the NBM cholinergic phenotype, but also a useful model in the assessment of regenerative protocols based on an efficient generation of stem cell-derived NBM cholinergic neurons. Indeed, we show that hfNBMs, without any manipulation and via systemic injection, were able to determine a functional recovery in NBM-lesioned rat brain.

The hfNBM primary cultures clearly showed a prominent neuronal phenotype, with high levels of MAP2 transcripts, which was confirmed by the flow cytometry detection of almost 100% of MAP2-positive cells. In addition, hfNBMs abundantly express both the neural precursor marker nestin and the neuronal progenitor marker β tubulin III, indicating coexisting undifferentiated cells already committed towards a specific phenotype. This plasticity was expected in primary cultures of fetal origin (Sarchielli et al., 2014) and may be of great value to study mechanisms potentially interfering with cholinergic neuron maturation and function. The hfNBM identity as cholinergic neurons was demonstrated by the expression of the major components of the cholinergic system, including proteins important for Ach synthesis, transport and hydrolysis (ChAT, VAchT and AchE, respectively). Accordingly, a basal release of Ach by these cells has been detected. In addition, the hfNBM neurons express CALB1 along with both high- (TrkA) and low- (p75/CD271) affinity NGF receptors, which altogether define the regional specificity of NBM cholinergic neurons (Abreu-Villaça et al., 2011). Indeed, in humans all the BF cholinergic neurons were also CALB1-positive (Geula et al., 1993) and co-expression of CALB1 with both NGF receptors is consistent with the pattern described for the Ch4 neurons of the human brain, which correspond to NBM according to the Mesulam’s nomenclature (Mesulam, 2013). Although, we might expect to also isolate GABAergic and glutamatergic neurons from the BF, in our primary cultures the majority of cells, as detected by flow cytometry, were ChAT-positive (97%). Our findings are in accordance with previous immunocytochemical and hybridization studies (Mufson et al., 2003) reporting a high enrichment of cholinergic neurons (90%) within the human NBM/Ch4 region.

The functional validation of the neuronal profile of hfNBM cells was provided by electrophysiological measurements, which demonstrated the presence of a polarized membrane potential (~−45 mV), the expression of functional, TTX-sensitive, voltage-gated Na^+^ channels (I_Na_), along with the presence of active phenomena of firing activity, in line with previous data on human developing neuronal cells (Lepski et al., 2011; Song et al., 2013). Furthermore, we detected transient I_A_ currents, delayed rectifier I_K_ and inwardly rectifying K_ir_ currents. The presence of I_A_ and I_K_ currents has been previously detected in human fetal brain neurons (Sah, 1995). In our population of isolated hfNBM neurons, I_K_ were the predominant conductance recorded in all cells tested and I_Na_ currents were present in the vast majority (89%) of hfNBMs. Transient I_A_ currents and Kir conductances were only detected in a subset of recorded cells (37% and 41%, respectively), suggesting that hfNBMs culture might include a heterogeneous population of neurons at different maturation steps or with distinct arrays of K^+^ channels. Interestingly, hfNBMs also exhibited the presence of functional cholinergic receptors. Ach binds to two distinct types of receptors: ionotropic nicotinic (nAchR) and metabotropic muscarinic (mAchR) receptors, located in both pre- and post-synaptic membranes of neurons. In particular, nAchR are pentameric ligand-gated ion channels resulting from the combination of different α (α2–10) and β (β2–4) subunits (Gotti et al., 2007). We demonstrated not only that hfNBM cells expressed, at mRNA level, all nAchR subunits investigated, with the α5 subunit being the most abundant, but also that they were able to respond to nicotine by triggering a 0 mV-reverting ramp-evoked current and membrane depolarization. Our results are consistent with the early detection of nAChR proteins and gene transcripts in brains from human fetuses of 4–12 weeks of gestational age (Hellström-Lindahl et al., 1998). They also further support the important role proposed for nAchRs during brain development in modulating dendritic outgrowth, establishment of neuronal connections and synaptogenesis (Abreu-Villaça et al., 2011). Future investigations aimed at identifying the specific subunits assembly of nAchRs, which are active in hfNBMs in the different maturation states, will help to clarify the neurodevelopmental actions of Ach. The relative abundancy of the α5 subunit in hfNBMs may suggest the importance of this subunit in regulating the nAchRs activity during cholinergic neuron development. Indeed, among nAchRs subunits, the α5 plays a peculiar functional role, since it contributes to the lining of functionally unique nAchR channels, but only if co-expressed with both another alpha- and beta-type subunit (Ramirez-Latorre et al., 1996). Moreover, studies in animal models have shown that ubiquitously distributed presynaptic nAchRs serve as ligand-gated ion channels that promote neurotransmitter release (Wonnacott et al., 2006). Hence, further studies should be addressed to clarify if nAchRs in our cellular model of cholinergic neurons could play a role as presynaptic receptors recruited to regulate Ach release.

We also clearly show that Ach activates mAchRs receptors in hfNBMs. Five subtypes of mAchRs have been described (M1–M5) with an inhibitory (M2 and M4) or excitatory (M1, M3 and M5) action on neuronal excitability (Caulfield, 1993). M2 and M3 subtypes resulted the most abundant in hfNBMs. Again, our results are consistent with literature data highlighting the importance of mAchRs, like nAchRs, in the developing central nervous system linked to clear effects of Ach on neuronal proliferation, differentiation and survival (Abreu-Villaça et al., 2011). Moreover, the presence of abundant M2 transcripts along with specific M2-type binding sites may reflect a prominent regulatory role of mAchRs as autoreceptors on Ach release in hfNBM cells. In line with this observation, we describe an ATR-sensitive increase in delayed rectifier outward I_K_ currents upon Ach or Cch application, as known for Gi coupled metabotropic receptors such as M2 and M4 subtypes (Caulfield and Birdsall, 1998). This effect could be either due to a direct action of the G protein subunit/s on K^+^ channels, or by second messenger-mediated (i.e., cAMP-mediated) channel modulation, as both possibilities have been described in other cellular systems (Inanobe and Kurachi, 2014). Since either Ach or Cch effects on I_K_ currents peaked within only 1–2 min from agonist superfusion, we may hypothesize a direct action of the G_α_ or G_β/γ_ subunit/s coupled to muscarinic receptor activation on delayed rectifier K^+^ channels. Overall, it emerges from present data that nicotinic receptors excite hfNBMs by depolarizing the cell membrane and approaching them to firing threshold, whereas mAchR activation, by opening I_K_ channels, could result inhibitory on neuronal excitability. However, given the fetal origin of our cellular model, the specific recruitment of either nicotinic or muscarinic receptor subtypes could be related to maturation processes of the cholinergic phenotype induced by Ach, as reported (Bruel-Jungerman et al., 2011), rather than to specific functional responses, such as those occurring in the context of adult brain circuits. This issue merits further investigations in future studies.

Several lines of evidence indicate that NGF is an endogenous neurotrophic factor crucially required to support survival, maintenance, connectivity and function of the brain cholinergic neurons, as derived by extensive research involving a wide range of animal models (Dekker et al., 1991; Koliatsos et al., 1991). Consistently with these findings, we demonstrated that NGF induces, via TrkA activation and its downstream signaling pathways, proliferation, neuritogenesis and ChAT expression in hfNBMs, thus confirming also in a human model the role played by the neurotrophin in the development and maintenance of the cholinergic phenotype. Accordingly, phase 1 clinical trials based on NGF gene delivery to NBM proved that this approach has the potential to improve cognitive symptoms and modify neurological disease progression in AD patients (Tuszynski et al., 2005). Interestingly, among the effects observed upon NGF treatment, the increased percentage of hfNBMs exhibiting a primary cilium appears of particular interest given the recent involvement of this organelle in the adult neurogenesis (Han et al., 2008). Primary cilia are non-motile, 4–8 μm long sensory antennas protruding from the surface of nearly all cells of the body, able to mediate the cellular response to extracellular signals growth and other factors (Armato et al., 2013). In particular, primary cilia have been detected in adult murine and rat neural stem/progenitor cells of the dentate gyrus’s subgranular zone (SGZ), which may produce new granule cells when they are needed for new memory encoding (Einstein et al., 2010). Accordingly, primary cilia of SGZ cells showed structural damages in transgenic AD mouse models (Rodríguez et al., 2008). Nevertheless, the precise role that primary cilia play in the overall neuronal function is still unknown. Our results strongly suggest for the first time that primary cilia may be involved in the NGF-driven maturation of human NBM cholinergic neurons. Further investigations may clarify the effective role of the primary cilium in the signaling mechanisms directly or indirectly recruited by NGF in the overall neuronal maturation. Since most evidence suggested that primary cilia signaling is based on an enrichment of receptors on its membrane (Händel et al., 1999), it would be interesting to analyze whether NGF receptors co-localize to primary cilia of hfNBMs. In this regard, the p75 low-affinity NGF receptor has been found in adult murine hippocampal dentate gyrus granule cells (Chakravarthy et al., 2010) and showed to be involved in the amyloid β_42_ toxic activity (Perini et al., 2002).

Accumulating evidence in animal models indicated that various neuronal functions, including learning and memory processes, are beneficially influenced by estrogens (Engler-Chiurazzi et al., 2016). Accordingly, in humans, it has been reported that the risk of AD is higher for women than men, especially in post-menopausal women compared to their male counterpart, clearly suggesting a neuroprotective role of estrogens (Breitner et al., 1988; Jorm and Jolley, 1998). Indeed, a recent meta-analysis of modifiable risk factors for AD identified the use of estrogens among the protective factors (Xu et al., 2015). Although controversial literature exists about the effectiveness of estrogen replacement therapy in preventing AD, several animal studies have shown that estrogens are neuroprotective for multiple targets in the central nervous system, including hippocampal neurogenesis and BF cholinergic neuron survival and function (Engler-Chiurazzi et al., 2016). In this regard, we report that hfNBMs express estrogen receptors. In particular, besides the classical nuclear ERα and ERβ, hfNBM cells also express the membrane-associated GPR30, a G protein-coupled receptor responsible for the rapid non-genomic actions of estrogens (Revankar et al., 2005). The most striking finding of our study is that the different types of estrogen receptors could be differently involved in mediating estrogenic action on growth, differentiation and maintaining of cholinergic phenotype in hfNBMs. Indeed, exposing cells to E2 induced cell proliferation, neuritogenesis and ChAT expression, with all effects mimicked by the exposure to G1, the selective GPR30 agonist, clearly implying the recruitment of rapid non-genomic mechanisms. However, pre-treatment with tamoxifen (an effective ERα and ERβ antagonist) counteracted all the estrogen-induced changes in hfNBMs, except for ChAT protein expression increase that was significantly prevented only in the presence of the specific GPR30 antagonist G15. Hence, GPR30 appears to be crucially required for the estrogenic action on maintenance of cholinergic phenotype in hfNBMs. Our findings are in good agreement with the recent evidences in experimental animals, which revealed that GPR30 is positioned on BF cholinergic neurons to mediate important estrogenic actions, such as effects on cognitive performance (Hammond and Gibbs, 2011) and Ach release in the hippocampus (Gibbs et al., 2014). A better understanding of the precise molecular mechanisms through which GPR30 regulates memory and cognition in humans will help to the development of more effective therapies for preventing and treating cognitive decline associated with AD.

Another important finding of our study is the demonstration that hfNBMs were able to improve cognitive functions when administered to a rat model of AD, obtained via the unilateral lesion of NBM. In particular, hfNBMs administration in NBM-lesioned rats determined a significant recovery of memory deficits. Memory is the product of dynamic interactions among multiple systems in the brain. Notably, working and spatial memory for the platform location appears to be mainly hippocampus-dependent as this brain area, which receives cholinergic inputs from the BF nuclei, is necessary for acquisition and retrieval of spatial information. Indeed, lesions of the medial septal and diagonal band cholinergic neurons result in deficits in the MWM task (Janis et al., 1998). However, memory performance in MWM is also affected by the NBM neuron activity resulting NBM lesion and stimulation in deficits and improvements, respectively, in the acquisition of spatial memory tasks (Lee et al., 2016). The step-down inhibitory avoidance memory depends mainly on the integrated activity of entorhinal, parietal cortex and CAI areas. Hence, lesions of the NBM, which provides the major cholinergic innervation to the entire cortical mantle, are mainly responsible for memory deficits in this task (Torres et al., 1994; Giovannini et al., 2015; Lee et al., 2016). Accordingly, our findings showing that spatial memory impairments in the MWM test of untreated NBM-lesioned animals recovered after 3 days of training indicate that NBM does not play a primary role in the acquisition of spatial memory task and that other cellular and molecular mechanisms underlie the spontaneous and hfNBM-induced spatial memory restoration. In contrast, memory deficits in the step-down inhibitory avoidance test of untreated NBM-lesioned animals persisted during the entire test, indicating that the memory disturbance in this task mainly reflects NBM activity.

We did not specifically analyze whether neuronal connections between donor and host neurons occurred, as well as no data about an improvement of cholinergic activity (Ach secretion) in brains from QA-lesioned/hfNBM-treated compared to QA-lesioned/untreated rats are provided. However, based on both the *in vitro* functional characterization and *in vivo* results, it is plausible that hfNBMs, at least for 3 weeks, retained their phenotype able to exert a trophic activity on the host injured cholinergic area. In addition, the fact that hfNBMs, intravenous administered, were detected in the NBM injected either with QA or saline and not in other brain areas not interested by the insult indicates that a damage of the blood brain barrier favors the migration of cells to injured brain sites. Although the manner in which injected neurons systemically reach the damaged brain area remains unknown, similar studies in models of neurodegenerative diseases demonstrated that intravenous route for stem cell transplantation is feasible and effective (Lee et al., 2005; Shen et al., 2010; Martínez-Morales et al., 2013). Most likely, the ability of migrating and penetrating to a specific site, as well as recapitulating specific functions, is related to an already committed phenotype and may be ascribed to the fetal origin of our cellular model, thus supporting the validity of such a model for cell-based therapy purposes. Indeed, as occurred for the assessment of cell-based therapy protocols for the treatment of PD (Grealish et al., 2014), human fetal neurons may serve as an important reference in terms of morphology, maturation, marker expression and overall phenotypic properties.

In conclusion, this study is the first in which human NBM cholinergic neurons are isolated and fully characterized and where their potential role for functional repair, without any manipulation, has been tested *in vivo* in an experimental model of AD. Such a model of young neurons, clearly programmed to the cholinergic function, possesses functional channels and receptors able to respond to important physiological regulators (Ach, NGF, estrogens). It therefore represents an excellent tool to study the ontogenetic mechanisms regulating the development and maintenance of the human BF cholinergic system. In addition, this cellular model could be useful to assess new lines of research including disease modeling, cell-based therapy and drug screening in the field of neurodegenerative disorders.

## Author Contributions

AM conceived and carried out experiments, interpreted the results and wrote the manuscript. ES and GG contributed to the cell culture isolation, characterization and *in vitro* experiments. EC and AMP performed the electrophysiological analysis. DP, PN and FC contributed to the *in vivo* experiments and performed behavioral tests. PC contributed to gene expression experiments. LB and BM contributed to cell phenotype characterization. SA and GC contributed to the *in vitro* experiments, RM and RV performed binding experiments. SB contributed to the fetal human tissue collection. MM and PG contributed to the interpretation of the results and revised the manuscript. GBV contributed to the conception of the project and the interpretation of results, and wrote the manuscript. All authors have approved the final version of this manuscript, agree to be accountable for all aspects of the work and qualify for authorship.

## Conflict of Interest Statement

The authors declare that the research was conducted in the absence of any commercial or financial relationships that could be construed as a potential conflict of interest.

## References

[B1] Abreu-Villaça Y. Filgueiras C. C. Manhães A. C. (2011). Developmental aspects of the cholinergic system. Behav. Brain Res. 221, 367–378. 10.1016/j.bbr.2009.12.049 20060019

[B2] Ambrosini S. Sarchielli E. Comeglio P. Porfirio B. Gallina P. Morelli A.. (2015). Fibroblast growth factor and endothelin-1 receptors mediate the response of human striatal precursor cells to hypoxia. Neuroscience 289, 123–133. 10.1016/j.neuroscience.2014.12.073 25595970

[B3] Armato U. Chakravarthy B. Pacchiana R. Whitfield J. F. (2013). Alzheimer’s disease: an update of the roles of receptors, astrocytes and primary cilia (review). Int. J. Mol. Med. 31, 3–10. 10.3892/ijmm.2012.1162 23124509

[B4] Bartolini L. Casamenti F. Pepeu G. (1996). Aniracetam restores object recognition impaired by age, scopolamine, and nucleus basalis lesions. Pharmacol. Biochem. Behav. 53, 277–283. 10.1016/0091-3057(95)02021-7 8808132

[B5] Bissonnette C. J. Lyass L. Bhattacharyya B. J. Belmadani A. Miller R. J. Kessler J. A. (2011). The controlled generation of functional basal forebrain cholinergic neurons from human embryonic stem cells. Stem Cells 29, 802–811. 10.1002/stem.626 21381151 PMC3107131

[B6] Björklund A. Lindvall O. (2000). Cell replacement therapies for central nervous system disorders. Nat. Neurosci. 3, 537–544. 10.1038/75705 10816308

[B7] Breitner J. C. Silverman J. M. Mohs R. C. Davis K. L. (1988). Familial aggregation in Alzheimer’s disease: comparison of risk among relatives of early-and late-onset cases and among male and female relatives in successive generations. Neurology 38, 207–212. 10.1212/WNL.38.2.207 3340281

[B8] Bruel-Jungerman E. Lucassen P. J. Francis F. (2011). Cholinergic influences on cortical development and adult neurogenesis. Behav. Brain Res. 221, 379–388. 10.1016/j.bbr.2011.01.021 21272598

[B9] Caulfield M. P. (1993). Muscarinic receptors—characterization, coupling and function. Pharmacol. Ther. 58, 319–379. 10.1016/0163-7258(93)90027-b 7504306

[B10] Caulfield M. P. Birdsall N. J. (1998). International Union of Pharmacology. XVII. Classification of muscarinic acetylcholine receptors. Pharmacol. Rev. 50, 279–290. 9647869

[B11] Chakravarthy B. Gaudet C. Ménard M. Atkinson T. Chiarini A. Dal Prà I.. (2010). The p75 neurotrophin receptor is localized to primary cilia in adult murine hippocampal dentate gyrus granule cells. Biochem. Biophys. Res. Commun. 401, 458–462. 10.1016/j.bbrc.2010.09.081 20875398

[B12] Coppi E. Pedata F. Gibb A. J. (2012). P2Y_1_ receptor modulation of Ca^2+^-activated K^+^ currents in medium-sized neurons from neonatal rat striatal slices. J. Neurophysiol. 107, 1009–1021. 10.1152/jn.00816.2009 22131374 PMC3289470

[B13] Dekker A. J. Langdon D. J. Gage F. H. Thal L. J. (1991). NGF increases cortical acetylcholine release in rats with lesions of the nucleus basalis. Neuroreport 2, 577–580. 10.1097/00001756-199110000-00006 1756238

[B14] Einstein E. B. Patterson C. A. Hon B. J. Regan K. A. Reddi J. Melnikoff D. E.. (2010). Somatostatin signaling in neuronal cilia is critical for object recognition memory. J. Neurosci. 30, 4306–4314. 10.1523/JNEUROSCI.5295-09.2010 20335466 PMC3842454

[B15] Engler-Chiurazzi E. B. Singh M. Simpkins J. W. (2016). From the 90’s to now: a brief historical perspective on more than two decades of estrogen neuroprotection. Brain Res. 1633, 96–100. 10.1016/j.brainres.2015.12.044 26740397 PMC4762740

[B16] Ferreira-Vieira T. H. Guimaraes I. M. Silva F. R. Ribeiro F. M. (2016). Alzheimer’s disease: targeting the cholinergic system. Curr. Neuropharmacol. 14, 101–115. 10.2174/1570159x13666150716165726 26813123 PMC4787279

[B17] Gallina P. Paganini M. Lombardini L. Saccardi R. Marini M. De Cristofaro M. T.. (2008). Development of human striatal anlagen after transplantation in a patient with Huntington’s disease. Exp. Neurol. 213, 241–244. 10.1016/j.expneurol.2008.06.003 18601923

[B18] Geula C. Schatz C. R. Mesulam M. M. (1993). Differential localization of NADPH-diaphorase and calbindin-D28k within the cholinergic neurons of the basal forebrain, striatum and brainstem in the rat, monkey, baboon and human. Neuroscience 54, 461–476. 10.1016/0306-4522(93)90266-i 8336832

[B19] Gibbs R. B. Nelson D. Hammond R. (2014). Role of GPR30 in mediating estradiol effects on acetylcholine release in the hippocampus. Horm. Behav. 66, 339–345. 10.1016/j.yhbeh.2014.06.002 24928571 PMC4131743

[B20] Giovannini M. G. Lana D. Pepeu G. (2015). The integrated role of ACh, ERK and mTOR in the mechanisms of hippocampal inhibitory avoidance memory. Neurobiol. Learn. Mem. 119, 18–33. 10.1016/j.nlm.2014.12.014 25595880

[B21] González-Martínez D. Kim S. H. Hu Y. Guimond S. Schofield J. Winyard P.. (2004). Anosmin-1 modulates fibroblast growth factor receptor 1 signaling in human gonadotropin-releasing hormone olfactory neuroblasts through a heparan sulfate-dependent mechanism. J. Neurosci. 24, 10384–10392. 10.1523/JNEUROSCI.3400-04.2004 15548653 PMC6730313

[B22] Gorry J. D. (1963). Studies on the comparative anatomy of the Ganglion Basale of Meynert. Acta Anat. 55, 51–104. 10.1159/000142464 14101383

[B23] Gotti C. Moretti M. Gaimarri A. Zanardi A. Clementi F. Zoli M. (2007). Heterogeneity and complexity of native brain nicotinic receptors. Biochem. Pharmacol. 74, 1102–1111. 10.1016/j.bcp.2007.05.023 17597586

[B24] Grealish S. Diguet E. Kirkeby A. Mattsson B. Heuer A. Bramoulle Y.. (2014). Human ESC-derived dopamine neurons show similar preclinical efficacy and potency to fetal neurons when grafted in a rat model of Parkinson’s disease. Cell Stem Cell 15, 653–665. 10.1016/j.stem.2014.09.017 25517469 PMC4232736

[B25] Grossi C. Francese S. Casini A. Rosi M. C. Luccarini I. Fiorentini A.. (2009). Clioquinol decreases amyloid-β burden and reduces working memory impairment in a transgenic mouse model of Alzheimer’s disease. J. Alzheimers Dis. 17, 423–440. 10.3233/JAD-2009-1063 19363260

[B26] Grossi C. Rigacci S. Ambrosini S. Ed Dami T. Luccarini I. Traini C.. (2013). The polyphenol oleuropein aglycone protects TgCRND8 mice against Aß plaque pathology. PLoS One 8:e71702. 10.1371/journal.pone.0071702 23951225 PMC3738517

[B27] Hammond R. Gibbs R. B. (2011). GPR30 is positioned to mediate estrogen effects on basal forebrain cholinergic neurons and cognitive performance. Brain Res. 1379, 53–60. 10.1016/j.brainres.2010.11.098 21138734 PMC3046317

[B28] Han Y. G. Spassky N. Romaguera-Ros M. Garcia-Verdugo J. M. Aguilar A. Schneider-Maunoury S.. (2008). Hedgehog signaling and primary cilia are required for the formation of adult neural stem cells. Nat. Neurosci. 11, 277–284. 10.1038/nn2059 18297065

[B29] Händel M. Schulz S. Stanarius A. Schreff M. Erdtmann-Vourliotis M. Schmidt H.. (1999). Selective targeting of somatostatin receptor 3 to neuronal cilia. Neuroscience 89, 909–926. 10.1016/s0306-4522(98)00354-6 10199624

[B30] Hellström-Lindahl E. Gorbounova O. Seiger A. Mousavi M. Nordberg A. (1998). Regional distribution of nicotinic receptors during prenatal development of human brain and spinal cord. Dev. Brain Res. 108, 147–160. 10.1016/s0165-3806(98)00046-7 9693793

[B31] Inanobe A. Kurachi Y. (2014). Membrane channels as integrators of G-protein-mediated signaling. Biochim. Biophys. Acta 1838, 521–531. 10.1016/j.bbamem.2013.08.018 24028827

[B32] Janis L. S. Glasier M. M. Fulop Z. Stein D. G. (1998). Intraseptal injections of 192 IgG saporin produce deficits for strategy selection in spatial-memory tasks. Behav. Brain Res. 90, 23–34. 10.1016/s0166-4328(97)00078-8 9520211

[B33] Jorm A. F. Jolley D. (1998). The incidence of dementia: a meta-analysis. Neurology 51, 728–733. 10.1212/WNL.51.3.728 9748017

[B34] Kilimann I. Grothe M. Heinsen H. Alho E. J. Grinberg L. Amaro E. Jr.. (2014). Subregional basal forebrain atrophy in Alzheimer’s disease: a multicenter study. J. Alzheimers Dis. 40, 687–700. 10.3233/JAD-132345 24503619 PMC4120953

[B35] Koliatsos V. E. Clatterbuck R. E. Nauta H. J. Knüsel B. Burton L. E. Hefti F. F.. (1991). Human nerve growth factor prevents degeneration of basal forebrain cholinergic neurons in primates. Ann. Neurol. 30, 831–840. 10.1002/ana.410300613 1789695

[B36] Kostović I. (1986). Prenatal development of nucleus basalis complex and related fiber systems in man: a histochemical study. Neuroscience 17, 1047–1077. 10.1016/0306-4522(86)90077-1 3714039

[B37] Kračun I. Rösner H. (1986). Early Cytoarchitectonic development of the anlage of the basal nucleus of Meynert in the human fetus. Int. J. Dev. Neurosci. 4, 143–149. 10.1016/0736-5748(86)90039-0 3455579

[B39] Lee S. T. Chu K. Park J. E. Lee K. Kang L. Kim S. U.. (2005). Intravenous administration of human neural stem cells induces functional recovery in Huntington’s disease rat model. Neurosci. Res. 52, 243–249. 10.1016/j.neures.2005.03.016 15896865

[B38] Lee J. E. Jeong D. U. Lee J. Chang W. S. Chang J. W. (2016). The effect of nucleus basalis magnocellularis deep brain stimulation on memory function in a rat model of dementia. BMC Neurol. 16:6. 10.1186/s12883-016-0529-z 26757896 PMC4711102

[B40] Lepski G. Maciaczyk J. Jannes C. E. Maciaczyk D. Bischofberger J. Nikkhah G. (2011). Delayed functional maturation of human neuronal progenitor cells *in vitro*. Mol. Cell. Neurosci. 47, 36–44. 10.1016/j.mcn.2011.02.011 21362477

[B41] Liu Y. Weick J. P. Liu H. Krencik R. Zhang X. Ma L.. (2013). Medial ganglionic eminence-like cells derived from human embryonic stem cells correct learning and memory deficits. Nat. Biotechnol. 31, 440–447. 10.1038/nbt.2565 23604284 PMC3711863

[B42] Martínez-Morales P. L. Revilla A. Ocaña I. González C. Sainz P. McGuire D.. (2013). Progress in stem cell therapy for major human neurological disorders. Stem Cell Rev. 9, 685–699. 10.1007/s12015-013-9443-6 23681704

[B43] Matucci R. Nesi M. Martino M. V. Bellucci C. Manetti D. Ciuti E.. (2016). Carbachol dimers as homobivalent modulators of muscarinic receptors. Biochem. Pharmacol. 108, 90–101. 10.1016/j.bcp.2016.03.012 26996304

[B44] Mesulam M. M. (2013). Cholinergic circuitry of the human nucleus basalis and its fate in Alzheimer’s disease. J. Comp. Neurol. 521, 4124–4144. 10.1002/cne.23415 23852922 PMC4175400

[B45] Morelli A. Comeglio P. Filippi S. Sarchielli E. Vignozzi L. Maneschi E.. (2013). Mechanism of action of phosphodiesterase type 5 inhibition in metabolic syndrome-associated prostate alterations: an experimental study in the rabbit. Prostate 73, 428–441. 10.1002/pros.22584 22996758

[B46] Morelli A. Marini M. Mancina R. Luconi M. Vignozzi L. Fibbi B.. (2008). Sex steroids and leptin regulate the “first Kiss” (KiSS 1/G-protein-coupled receptor 54 system) in human gonadotropin-releasing-hormone-secreting neuroblasts. J. Sex. Med. 5, 1097–1113. 10.1111/j.1743-6109.2008.00782.x 18331266

[B47] Mufson E. J. Ginsberg S. D. Ikonomovic M. D. DeKosky S. T. (2003). Human cholinergic basal forebrain: chemoanatomy and neurologic dysfunction. J. Chem. Neuroanat. 26, 233–242. 10.1016/s0891-0618(03)00068-1 14729126

[B48] Paganini M. Biggeri A. Romoli A. M. Mechi C. Ghelli E. Berti V.. (2014). Fetal striatal grafting slows motor and cognitive decline of Huntington’s disease. J. Neurol. Neurosurg. Psychiatry 85, 974–981. 10.1136/jnnp-2013-306533 24347577 PMC4145428

[B49] Paxinos G. Watson C. (2006). The Rat Brain in Stereotaxic Coordinates. London: Academic Press.

[B50] Perini G. Della-Bianca V. Politi V. Della Valle G. Dal-Pra I. Rossi F.. (2002). Role of p75 neurotrophin receptor in the neurotoxicity by β-amyloid peptides and synergistic effect of inflammatory cytokines. J. Exp. Med. 195, 907–918. 10.1084/jem.20011797 11927634 PMC2193732

[B51] Pombero A. Bueno C. Saglietti L. Rodenas M. Guimera J. Bulfone A.. (2011). Pallial origin of basal forebrain cholinergic neurons in the nucleus basalis of Meynert and horizontal limb of the diagonal band nucleus. Development 138, 4315–4326. 10.1242/dev.069534 21865321

[B52] Ramirez-Latorre J. Yu C. R. Qu X. Perin F. Karlin A. Role L. (1996). Functional contributions of α5 subunit to neuronal acetylcholine receptor channels. Nature 380, 347–351. 10.1038/380347a0 8598930

[B53] Revankar C. M. Cimino D. F. Sklar L. A. Arterburn J. B. Prossnitz E. R. (2005). A transmembrane intracellular estrogen receptor mediates rapid cell signaling. Science 307, 1625–1630. 10.1126/science.1106943 15705806

[B54] Rodríguez J. J. Jones V. C. Tabuchi M. Allan S. M. Knight E. M. LaFerla F. M.. (2008). Impaired adult neurogenesis in the dentate gyrus of a triple transgenic mouse model of Alzheimer’s disease. PLoS One 3:e2935. 10.1371/journal.pone.0002935 18698410 PMC2492828

[B55] Sah D. W. (1995). Human fetal central neurons in culture: voltage- and ligand-gated currents. J. Neurophysiol. 74, 1889–1899. 8592182 10.1152/jn.1995.74.5.1889

[B56] Sarchielli E. Comeglio P. Squecco R. Ballerini L. Mello T. Guarnieri G.. (2017). Tumor necrosis factor-α impairs kisspeptin signaling in human gonadotropin-releasing hormone primary neurons. J. Clin. Endocrinol. Metab. 102, 46–56. 10.1210/jc.2016-2115 27736314 PMC5413096

[B57] Sarchielli E. Marini M. Ambrosini S. Peri A. Mazzanti B. Pinzani P.. (2014). Multifaceted roles of BDNF and FGF2 in human striatal primordium development. An *in vitro* study. Exp. Neurol. 257, 130–147. 10.1016/j.expneurol.2014.04.021 24792640

[B58] Shen C. C. Lin C. H. Yang Y. C. Chiao M. T. Cheng W. Y. Ko J. L. (2010). Intravenous implanted neural stem cells migrate to injury site, reduce infarct volume, and improve behavior after cerebral ischemia. Curr. Neurovasc. Res. 7, 167–179. 10.2174/156720210792231822 20560882

[B59] Song M. Mohamad O. Chen D. Yu S. P. (2013). Coordinated development of voltage-gated Na^+^ and K^+^ currents regulates functional maturation of forebrain neurons derived from human induced pluripotent stem cells. Stem Cells Dev. 22, 1551–1563. 10.1089/scd.2012.0556 23259973 PMC3653388

[B60] Torres E. M. Perry T. A. Blockland A. Wilkinson L. S. Wiley R. G. Lappi D. A.. (1994). Behavioural, histochemical and biochemical consequences of selective immunolesions in discrete regions of the basal forebrain cholinergic system. Neuroscience 63, 95–122. 10.1016/0306-4522(94)90010-8 7898665

[B61] Tuszynski M. H. Thal L. Pay M. Salmon D. P. U H. S. Bakay R.. (2005). A phase 1 clinical trial of nerve growth factor gene therapy for Alzheimer disease. Nat. Med. 11, 551–555. 10.1038/nm1239 15852017

[B62] Wonnacott S. Barik J. Dickinson J. Jones I. W. (2006). Nicotinic receptors modulate transmitter cross talk in the CNS: nicotinic modulation of transmitters. J. Mol. Neurosci. 30, 137–140. 10.1385/jmn:30:1:137 17192660

[B63] Xu Q. Tam M. Anderson S. A. (2008). Fate mapping Nkx2.1-lineage cells in the mouse telencephalon. J. Comp. Neurol. 506, 16–29. 10.1002/cne.21529 17990269

[B64] Xu W. Tan L. Wang H. F. Jiang T. Tan M. S. Tan L.. (2015). Meta-analysis of modifiable risk factors for Alzheimer’s disease. J. Neurol. Neurosurg. Psychiatry 86, 1299–1306. 10.1136/jnnp-2015-310548 26294005

[B65] Yue W. Li Y. Zhang T. Jiang M. Qian Y. Zhang M.. (2015). ESC-derived basal forebrain cholinergic neurons ameliorate the cognitive symptoms associated with Alzheimer’s disease in mouse models. Stem Cell Reports 5, 776–790. 10.1016/j.stemcr.2015.09.010 26489896 PMC4649256

