# Supplementary material for: Young Human Cholinergic Neurons Respond to Physiological Regulators and Improve Cognitive Symptoms in an Animal Model of Alzheimer’s Disease
Source: Front Cell Neurosci. 2017 Oct 27;11:339. doi: 10.3389/fncel.2017.00339 (PMC5666298; doi:10.3389/fncel.2017.00339)
Supplement: Supplementary file 1 [file Data_Sheet_1.doc]

Supplementary Material

**Young Human Cholinergic Neurons Respond To Physiological Regulators And Improve Cognitive Symptoms In An Animal Model Of Alzheimer's Disease**

Annamaria Morelli^*^, Erica Sarchielli, Giulia Guarnieri, Elisabetta Coppi, Daniela Pantano, Paolo Comeglio, Pamela Nardiello, Anna Maria Pugliese, Lara Ballerini, Rosanna Matucci, Stefano Ambrosini, Giuseppe Castronovo, Rosa Valente, Benedetta Mazzanti, Sandra Bucciantini, Mario Maggi, Fiorella Casamenti, Pasquale Gallina, Gabriella B. Vannelli

*** Correspondence:** Annamaria Morelli: [a.morelli@unifi.it](mailto:a.morelli@unifi.it)

1. **Detailed Methods**
   1. **Cell culture**

The use of human fetal tissue for research purposes was approved by the National Ethics Committee and the local ethic committee for investigation in Humans of the University of Florence (Permit Number: 678304). Human fetuses biopsies were obtained from therapeutic medical abortions after women approved and signed the informed consent document, as already reported (Gallina et al., 2008). NBM tissue was dissected from two female 12 weeks old human fetuses under sterile conditions, cut into fragments and enzymatically digested by 1 mg/ml collagenase type IV (Sigma-Aldrich Corp., St. Louis, MO, USA) incubation. The cell suspensions were mechanically dispersed by pipetting in Coon's modified Ham's F12 medium (Euroclone, Milan, Italy) supplemented with 10% FBS (Hyclone, Logan, UT) and cultured at 37°C in 5% CO_2_ atmosphere. Confluent cells were split to 1:2-1:4 ratio using EDTA–trypsin solution, and used within the 26^th^ passage.

- 1. **RNA extraction and quantitative real time RT-PCR**

Isolation of total RNA from cells was performed using the “RNeasy Micro kit” (Qiagen, Hilden, Germany) according to the manufacturers' instructions. cDNA synthesis was carried out using the iScript^TM^ cDNA Synthesis Kit purchased from Bio-Rad Laboratories (Hercules, CA, USA). For some genes, quantitative real time RT-PCR (qRT-PCR) was performed according to the fluorescent TaqMan methodology, as previously described (Morellli et al., 2008). Primers and probes for the target genes were predeveloped assays (Life Technologies, Carlsbad, CA, USA) as listed in supplementary Table 1. For the remaining genes, qRT-PCR was performed using SsoFast^TM^ EvaGreen® Supermix (Bio-Rad Laboratories) as previously described (Morelli et al., 2013). Specific primers sequences for the target genes are reported in supplementary Table 2 The expression of the 18S ribosomal RNA subunit, chosen as the housekeeping gene, was quantified with a predeveloped assay (Hs99999901_s1; Life Technologies) and used for relative quantification of the target genes. Data analysis was based on the comparative threshold cycle (Ct) using the 2^−ΔΔCt^ method as previously described (Livak and Schmittgen, 2001). Amplification and detection were performed with the MyIQ2^TM^ Two-Color Real-Time PCR Detection System (Bio-Rad Laboratories).

- 1. **Immunofluorescence**

Immunofluorescence analysis was performed as previously described (Sarchielli et al., 2014). Briefly, the cells grown on sterile slides were fixed in 2% paraformaldehyde in phosphate buffered saline (PBS) for 15’ at room temperature, followed by permeabilization in PBS containing 0.1% Triton X-100 for 15’. Immunostaining was performed using the following primary antibodies: anti-ChAT pAb (1:200; Millipore, Temecula, CA, USA), anti-VAchT pAb (1:1000, Sigma-Aldrich Corp.), anti-GFAP mAb (1:100; Santa Cruz Biotechnology, Santa Cruz, CA, USA), anti-α tubulin mAb (1:2000, Santa Cruz Biotechnology), anti-acetylated α-tubulin mAb (1:500, Sigma-Aldrich Corp.), anti-ERα mAb (1:50, Santa Cruz Biotechnology), anti-ERβ mAb (1:50, Santa Cruz Biotechnology), anti-GPR30 pAb (1:40, Abcam), followed by Alexa Fluor 488 or 568 goat anti-rabbit or Alexa Fluor 488 goat anti-mouse (1:200, Molecular Probes, Eugene, Oregon, USA), as appropriate. For α-tubulin and acetylated α-tubulin staining, cells were cultured in serum/phenol red-free condition and treated with NGF (100 ng/ml), E2 (10 nM) or G1 (100 nM) for 24/48 h in presence or absence of the receptor inhibitors K252a (200 nM), Tamoxifen (100 nM) or G15 (1 μM), respectively. The number of cells with neurites longer than four times the cell body (α-tubulin staining) or the number of ciliated cells (acetylated α-tubulin staining) was calculated by counting the stained cells in ten fields per slide of three different experiments performed in triplicate.

- 1. **Flow cytometry**

hfNBMs were analyzed by flow cytometry as previously described (Urbani et al., 2006; Sarchielli et al., 2017). Briefly, aliquots of cells were resuspended in buffer (PBS, 1% FBS) and incubated with the following primary antibodies: anti-O4 mAb (1:100, Millipore), anti-GFAP mAb (1:100, Santa Cruz Biotechnology), anti-MAP2 pAb (1:100, Millipore), anti-ChAT pAb (1:100, Millipore), anti-TrkA pAb (1:100, Santa Cruz Biotechnology). In particular, the surface oligodendrocyte marker O4 was assessed by performing a first incubation with the primary antibody followed by incubation with Alexa Fluor 488 goat anti-mouse IgM (1:200, Molecular Probes). For the other markers, cells were first incubated with paraformaldehyde 3.7% for 15′ at room temperature, washed with buffer (PBS, 1% FBS) and incubated with Triton X-100 0.02% for 30′ at room temperature; finally cells were washed twice with cold buffer and stained with the primary antibodies. Alexa Fluor 568 goat anti-rabbit or Alexa Fluor 488 goat anti-mouse IgG (H+L) (1:200, Molecular Probes) were used as secondary antibodies. Cells were analyzed on a FACSCanto II instrument (BD Pharmingen, San Diego, CA, USA). Each area of positivity was determined by gating on the same cells stained with isotype-matched mAbs. Data were analyzed using BD FACSDiva Software (BD) and FlowJo v10 (Tree Star, Inc., Ashland, OR, USA).

- 1. **Acetylcholine release assay**

Cells (8x10^5)^ were seeded in 60mm diameter plates in culture medium supplemented with 10% FBS and grown until subconfluence. Then the cells were maintained in choline chloride free medium (Sigma-Aldrich Corp.) for additional 24 h before collecting the culture medium. The Ach release was immediately quantified by Choline/Acetylcholine Assay Kit (Abcam, Cambridge, UK), according to the manufacturer’s instructions. The measurements were performed with a FlexStation 3 Microplate Reader (Molecular Devices, Sunnyvale, CA, USA).

- 1. **Western Blot Analysis**

Cells were cultured in serum/phenol red-free condition and treated with NGF (10-100 ng/ml), E2 (10^-10^-10^-7^ M) or G1 (100 nM) in presence or absence of the receptor inhibitors K252a (200 nM), Tamoxifen (100 nM) or G15 (1 μM). Protein extracts were obtained in standard lysis buffer supplemented with protease and phosphatase inhibitor cocktails (Sigma-Aldrich Corp.); protein concentration was measured with Coomassie protein assay kit (Bio-Rad Laboratories) and aliquots containing 20 μg of proteins were loaded on SDS-PAGE. Proteins were then transferred on polyvinylidene difluoride membranes (GE Healthcare, Little Chalfont, UK), blocked in 3% BSA and incubated with the following primary antibodies: anti-p-TrkA pAb (1:1000, Cell Signaling Technologies, Danvers, MA, USA), anti-pERK 1/2 mAb (1:1000, Cell Signaling Technologies), anti-p-CREB pAb (1:1000, Santa Cruz Biotechnology), anti-c fos pAb (1:1000, Sigma-Aldrich), anti-α-tubulin mAb (1:2000, Santa Cruz Biotechnology), anti-ChAT pAb (1:2000, Millipore), anti-β actin mAb (1:10000, Santa Cruz Biotechnology), anti-GAP43 mAb (1:1000, Santa Cruz Biotechnology), anti-STAT1 pAb (1:1000, Santa Cruz Biotechnology). The incubation with the primary antibodies was followed by peroxidase conjugated secondary IgG treatment (Santa Cruz Biotechnology) and the reacted proteins were revealed by the enhanced chemiluminescence system (Euroclone). Image acquisition and densitometric analysis were performed with Quantity One software on a ChemiDoc XRS instrument (Bio-Rad Laboratories Inc.) and using α-tubulin or β actin for normalization.

- 1. **Electrophysiology**

Whole-cell patch-clamp recordings were performed on hfNBMs from p14 to p26. Cells grown on a poly-L-lisine-coated glass coverslip were transferred to a small chamber mounted on the platform of an inverted microscope (Olympus CKX41, Milan, Italy) and superfused at a flow rate of 2 ml/min. Borosilicate glass electrodes (Harvard Apparatus, Holliston, MA, USA) were pulled with a Sutter Instruments puller (model P-87) to a final tip resistance of 4–7 MΩ. All the experiments were carried out at RT (20–22 °C). Capacitive transients generated by the electrode and by cell membrane were digitally subtracted by the amplifier circuit. Series resistance (Rs), Rm and Cm were routinely measured by fast hyperpolarizing voltage pulses (from −60 to −70 mV, 40 ms duration). Immediately after breakthrough into whole-cell configuration, cell Vm at rest was determined by switching to the current-clamp mode. A 800 ms voltage ramp protocol from +80 mV to −120 mV (in order to inactivate eventual I_Na_ currents) was recorded used to evoke a wide range of overall voltage-dependent membrane currents. Ramps were evoked continuously before, during and after drug application (with a 15 s interval). Variations of Vm induced by drug treatments were measured by calculating the reversal potential (the “zero current” potential) of ramp-evoked currents before, during and after drug application. Outward K^+^ currents were evoked by 13 depolarizing voltage steps (10 mV steps from −40 to +80 mV, 200 ms each, 1 s inter-step interval) preceded by a 60 ms pre-step (Vpre) at −80 mV in order to activate either delayed rectifier outward K^+^ currents (I_K_) or transient outward (I_A_) conductances. Current-to-voltage relationships (I–V plots) of I_K_ or I_A_ currents were obtained by measuring current amplitude at the steady state (200–250 ms after step onset) or as a peak (1–20 ms after step onset), respectively. Inward rectifying K^+^ (K_ir_) currents were activated by hyperpolarizing voltage steps (10 mV steps from −10 mV to −130 mV, 100 ms each, 1 s inter-step interval) with a Vpre of 0 mV (100 ms duration) in order to inactivate eventual inward Na^+^ currents (I_Na_). I_Na_ were activated by a depolarizing voltage step protocol (10 mV steps from −50 mV to +40 mV, 15 ms each, 2 s inter-step interval) starting from a holding potential (Vh) of −90 mV in order to remove Na^+^ channel inactivation in the presence or absence of 1µM TTX. To obtain the activation-inactivation curves of I_Na_, we monitored peak I_Na_ elicited by the following protocol: 18 depolarizing voltage steps (from -130 to + 50 mV, 40 ms duration, 2 s inter-step interval) were applied from a Vh = - 90 mV (peak I_Na_ measured at each step was used for the activation curve) and were followed by a 15 ms step at -10 mV (peak I_Na_ measured at each step was used for the inactivation curve). The normalized G-V curves were averaged and fitted by a Boltzmann equation: G/G_max_={1 + exp [(V_1/2_ – Vm)/*k*]}^-1^ (Yin et al., 2017). Current amplitude (measured as pA) was normalized to respective cell capacitance (measured in pF) and expressed as current density (pA/pF) in averaged results. Ach 10 µM (Sigma Aldrich Corp.), Cch 50 µM (Sigma Aldrich Corp.), ATR 100 nM (Sigma Aldrich Corp.), and TTX (Ascent Scientific, Cambridge, UK) were applied by superfusion with a three-way perfusion valve controller (Harvard Apparatus) after a stable baseline was obtained. A complete exchange of bath solution in the recording chamber was achieved within 30 s. Current-clamp recordings were performed by applying 12 steps of current injection (300 ms duration; 100 pA increment, from -100 to 1000 pA) from the resting membrane potential of the investigated cell, as already described (Coppi et al., 2012). Current-clamp recordings were ﬁltered at 10 kHz and digitized at 20 kHz. The rate of AP depolarization and hyperpolarization was measured as the ﬁrst derivative of membrane potential over time (mV/ms). AP threshold was deﬁned as the voltage at which the derivative first exceeded 30 mV/ms. AP amplitude was calculated as the difference between the peak reached by the overshoot and the threshold. AP half width was measured at half the amplitude. The fast after-hyperpolarization potential (fAHP) was measured as the difference between threshold and the minimum potential reached after the AP peak within 10 ms. No slow AHP (sAHP) after the end of the current step was observed in any cell tested. Voltage sag was estimated as the ratio of peak and steady-state voltages during 250 ms current step at -100 pA.

- 1. **Radioligand Equilibrium Binding Assays**

For cell membranes preparation, cells were harvested and then homogenized in binding buffer (PB, 25 mM sodium phosphate buffer, 5 mM MgCl_2_, pH 7.4) using an Ultra-Turrax tissue homogenizer (for 30 s, 16000 rpm). The homogenate was then sedimented by centrifugation (17000*g* for 15 min at 4 °C) and the pellet was resuspended in PB, rehomogenized with Ultra-Turrax for 30 s, aliquoted and stored frozen at -80°C until required for radioligand binding assays. Protein concentration was measured with Coomassie protein assay kit (Bio-Rad Laboratories) and radioligand binding experiments were performed as previously described with minor modifications (Matucci et al., 2016). Briefly, for saturation binding assays 50 μl of cell membranes (0.5-0.7 mg/ml) were incubated in 500 μl total volume of PB buffer containing concentrations of [^3^H]NMS (Perkin-Elmer Life and Analytical Science) ranging from 0.05 to 1.6 nM, for 2 hour at room temperature. Nonspecific binding was defined using 10 μM Atropine (Sigma-Aldrich). Incubation was terminated by rapid filtration through Whatman GF/B filters pre-soaked in a 0.05% polyethylenimine (PEI) solution for at least 1 hour, using a Brandel cell harvester (Brandel, Gaithersburg, MD, USA). Filters were washed three times with ice-cold milliQ water and dried before the addition of scintillation cocktail (Filter Count, Perkin Elmer Life and Analytical Science, Milano, Italy). Vials were then left to stand until the filters became uniformly translucent before radioactivity was determined using scintillation counting (TRI-CARB 1100, Perkin-Elmer Life and Analytical Science).

For competition binding assays, membranes fractions containing a fixed amount of protein (50 µl), 50 µl of 0.2 nM [^3^H]NMS, and 50 µl of increasing concentrations of the muscarinic antagonist metoctramine (0.1 nM- 0.1 mM; Sigma-Aldrich) were incubated as described above. The determination of nonspecific binding, termination of the reaction, and determination of bound radioactivity were performed as described above. In all instances, the amount of radioligand bound was less than 10% of the total amount added; data were thus analyzed using models that do not assume radioligand depletion. All measurements were obtained in duplicate.

- 1. **Cell proliferation assay**

Cell proliferation was determined by MTT assay (Sigma-Aldrich Corp.). Briefly, 9x10^3^ cells were seeded in 96-well plates in medium supplemented with 10% FBS. After 24 h cells were maintained in serum/phenol red-free medium for 8 h and subsequently incubated for 24 h in serum/phenol red-free medium containing NGF (10-100 ng/ml), E2 (10^-13^-10^-6^ M) or G1 (10^-9^-10^-6^ M) in presence or absence of the receptor inhibitors K252a 200 nM, tamoxifen 100 nM or G15 1µM. Then the medium was changed and 10 µl of MTT solution was added for 3 h at 37 °C. The optical density was measured at 450 nm using a Multiskan FC spectrophotometer (Thermo Fisher Scientific, Milan, Italy). Cell viability was expressed as relative percentage of viable cells over control, taken as 100% (mean±SEM) from three separate experiments performed in quadruplicate.

- 1. **In vivo study**
     1. **Animals**

Three-month-old, 230-250 g male Wistar rats (Harlan, Milan, Italy) (n=5-6 per group) were used. Animals were housed in macrolon cages with ad libitum food and water and maintained on a 12h light/dark cycle at 23°C. All experiments were carried out according to the EC Directive 86/609/EEC for animal experiments and National guidelines for animal care (Permit Number: 567/2015-PR; Italian Ministry of Health). All efforts were made to minimize the number of animals used and their suffering.

An injection into the right NBM of 0.5 µl of 0.12 M QA (Sigma-Aldrich Corp.), dissolved in 50 mM PBS pH 7.4 or 0.9% saline (control injection) was made by means of a Hamilton microsyringe at the following stereotactic coordinates: AP= -0.2; L= - 2.8 and H= 6.8 from Bregma (Paxinos and Watson, 2006) in anesthetized (chloral hydrate, 400 mg/kg, i.p., plus Carprofen, 5 mg/kg, subcutaneously) rats, as previously described (Baglioni et al., 2006). The animals were equally divided into four groups. Group I: rats injected into the NBM with 0.5 μl of QA, treated with Ciclosporine (2 mg/kg/day; Wennersten et al., 2006), one day prior the intravenous administration by the tail vein of 300 μl of 1.5x10^6^ hfNBMs and for all the length of the experiment. Rats were sacrificed on day 1, 7 and 21 after hfNBMs administration. Group II: rats injected into the NBM with 0.5 μl of QA and sacrificed 21 days after injection. Group III: rats injected into the NBM with 0.5 μl saline (control), treated with Cyclosporine (2 mg/kg/day), one day prior the intravenous. administration by the tail vein of 300 μl of 1.5 x10^6^ hfNBMs and for all the length of the experiment. Rats were sacrificed 21 days after hfNBMs administration. Group IV: controls, un-injected rats. Before administration the cells were labeled with the PKH26 Red Fluorescent dye (Sigma-Aldrich Corp) according to the manufacturer’s instructions.

- - 1. **Animal Tissue Processing**

Twenty-four hours, seven and twenty-one days after injection, rats were deeply anesthetized (chloral hydrate, 400 mg/kg ip,) and perfused transcardially with 0.9% saline followed by 4% paraformaldehyde in 0.1 M PBS (pH 7.4) at a flow rate of 10 ml/min for 30 min. After sacrifice, brains were quickly extracted and fixed in phosphate-buffered 4% paraformaldehyde (pH 7.4) for 48 h at 4°C. Subsequently brains were rinsed with PBS, dehydrated using an automated machine, and paraffin embedded. Coronal sections (5.0 μm) were cut using a microtome and mounted on slides. Livers from rats subjected to intravenous administration of hfNBMs were harvested and analyzed to detect the presence of PKH26 labeled cells in systemic organs.

- - 1. **Immunohistochemistry**

Immunohistochemical analyses were performed on 5.0 μm coronal paraffin-embedded sections, as previously described (Rosi et al., 2010). Anti-GFAP (1:1000; Agilent Technologies, Santa Clara, CA, USA) and anti-ChAT (1:200; Millipore) pAbs were used to detect astrocytes and cholinergic neurons, respectively. ChAT-positive cells in the NBM were counted under a 10 × objective lens, using an Olympus BX63 microscope. Five sections per animal, anteroposterior standardized with respect to the injection site and spaced 50–100 μm from one another, were analyzed. The total number of ChAT-positive cells in the QA injected NBM was averaged, expressed as a percentage of that counted in the saline-injected NBM, assumed as 100% (n=3 per group), and analyzed using Prism 5.0 (GraphPad Software, San Diego, CA, USA).

- - 1. **Morris Water Maze test**

During the third week after hfNBMs administration rats were tested in the Morris Water Maze (MWM) (n= 5-6 per group). The MWM apparatus consisted of a circular pool (1.6 m in diameter and 0.36 m high) made of green plastic. The pool was filled to a depth of 20 cm with water (24-25 °C) that was made dark by the addition of non-toxic dark paint. Rats were tested in the reference memory version of MWM with the procedure previously described for mice (Grossi et al., 2009).

- - 1. **Step-Down inhibitory avoidance task**

The day after the end of MWM task the animals were tested in the Step-Down inhibitory avoidance task. The apparatus was an open field plexiglas box (50 × 25 × 25 cm) with a steel rod floor and a plexiglas platform (5 × 8 × 25 cm) set on the grid floor to which intermittent electric shocks were delivered. The apparatus and procedures used for the “Step-Down” inhibitory avoidance test were previously described for mouse (Grossi et al., 2013) with some modifications.

- 1. **Statistical analysis**

Data are expressed as mean ± SEM. Student's paired or unpaired *t*-tests or One- and two-way ANOVA followed by Newman–Keuls or Bonferroni post-test analysis were performed, as appropriate, in order to determine statistical significance (set at p<0.05). Data were analyzed using software package GraphPad Prism (GraphPad Software).

1. **12. References to supplemental methods**

Ambrosini, S., Sarchielli, E., Comeglio, P., Porfirio, B., Gallina, P., Morelli, A., et al. (2015). Fibroblast growth factor and endothelin-1 receptors mediate the response of human striatal precursor cells to hypoxia*. Neuroscience.* 289, 123-33. doi: 10.1016/j.neuroscience.2014.12.073

Baglioni, S., Casamenti, F., Bucciantini, M., Luheshi, L.M., Taddei, N., Chiti, F., et al. (2006). Prefibrillar amyloid aggregates could be generic toxins in higher organisms. *J. Neurosci.* 26, 8160-8167. doi: 10.1523/JNEUROSCI.4809-05.2006

Gallina, P., Paganini, M., Lombardini, L., Saccardi, R., Marini, M., De Cristofaro, M.T., et al. (2008). Development of human striatal anlagen after transplantation in a patient with Huntington's disease. *Exp. Neurol.* 213, 241-244. doi: 10.1016/j.expneurol.2008.06.003

Grossi, C., Francese, S., Casini, A., Rosi, M.C., Luccarini, I., Fiorentini, A., et al. (2009). Clioquinol decreases amyloid-beta burden and reduces working memory impairment in a transgenic mouse model of Alzheimer's disease. *J. Alzheimers Dis.* 17, 423-440. doi: 10.3233/JAD-2009-1063.

Coppi, E., Pedata, F., and Gibb, A.J. (2012). P2Y1 receptor modulation of Ca2+-activated K+ currents in medium-sized neurons from neonatal rat striatal slices. J. Neurophysiol. 107, 1009-1021. doi: 10.1152/jn.00816.2009.

Grossi, C., Rigacci, S., Ambrosini, S., Ed Dami, T., Luccarini, I., Traini, C., et al. (2013). The polyphenol oleuropein aglycone protects TgCRND8 mice against Aß plaque pathology. *PLoS One* 8, e71702. doi: 10.1371/journal.pone.0071702. eCollection 2013.

Livak, K.J., and Schmittgen, T.D. (2001). Analysis of relative gene expression data using real-time quantitative PCR and the 2(−Delta Delta C(T)) Method. Methods 25, 402-408. doi: 10.1006/meth.2001.1262

Matucci, R., Nesi, M., Martino, M.V., Bellucci, C., Manetti, D., Ciuti, E., et al. (2016). Carbachol dimers as homobivalent modulators of muscarinic receptors. *Biochem. Pharmacol.* 108, 90-101.

Morelli, A., Comeglio, P., Filippi, S., Sarchielli, E., Vignozzi, L., Maneschi, E., et al. (2013). Mechanism of action of phosphodiesterase type 5 inhibition in metabolic syndrome-associated prostate alterations: an experimental study in the rabbit. *Prostate* 73, 428-441. doi: 10.1002/pros.22584

Morelli, A., Marini, M., Mancina, R., Luconi, M., Vignozzi, L., Fibbi, B., et al. (2008). Sex steroids and leptin regulate the "first Kiss" (KiSS 1/G-protein-coupled receptor 54 system) in human gonadotropin-releasing-hormone-secreting neuroblasts. *J. Sex. Med*. 5, 1097-1113. doi: 10.1111/j.1743-6109.2008.00782.x.

Paxinos, G., and Watson, C. (2006). *The Rat Brain in Stereotaxic Coordinates*. London: Academic Press.

Rosi, M.C., Luccarini, I., Grossi, C., Fiorentini, A., Spillantini, M.G., Prisco, A., et al. (2010). Increased Dickkopf-1 expression in transgenic mouse models of neurodegenerative disease. *J. Neurochem.* 112, 1539-1551. doi: 10.1111/j.1471-4159.2009.06566.x.

Sarchielli, E., Comeglio, P., Squecco, R., Ballerini, L., Mello, T., Guarnieri, G., et al. (2017). Tumor Necrosis Factor-α Impairs Kisspeptin Signaling in Human Gonadotropin-Releasing Hormone Primary Neurons. *J. Clin. Endocrinol. Metab*. 102, 46-56. doi: 10.1210/jc.2016-2115.

Sarchielli, E., Marini, M., Ambrosini, S., Peri, A., Mazzanti, B., Pinzani, P., et al. (2014). Multifaceted roles of BDNF and FGF2 in human striatal primordium development. An in vitro study. *Exp. Neurol.* 257, 130-147. doi: 10.1016/j.expneurol.2014.04.021.

Urbani, S., Caporale, R., Lombardini, L., Bosi, A., and Saccardi, R. (2006). Use of CFDA-SE for evaluating the in vitro proliferation pattern of human mesenchymal stem cells. *Cytotherapy* 8, 243-253.

Wennersten, A., Holmin, S., Al Nimer, F., Meije,r X., Wahlberg, L.U., and Mathiesen, T. (2006). Sustained survival of xenografted human neural stem/progenitor cells in experimental brain trauma despite discontinuation of immunosuppression. *Exp. Neurol.* 199, 339-347. doi: 10.1016/j.expneurol.2005.12.035

Yin, L., Rasch, M.J., He, Q., Wu, S., Dou, F., and Shu, Y. (2017). Selective Modulation of Axonal Sodium Channel Subtypes by 5-HT1A Receptor in Cortical Pyramidal Neuron. *Cerebral Cortex* 27, 509-521. doi: 10.1093/cercor/bhv245.

1. **Supplementary Figures and tables**
   1. **Supplementary Figure 1**

**
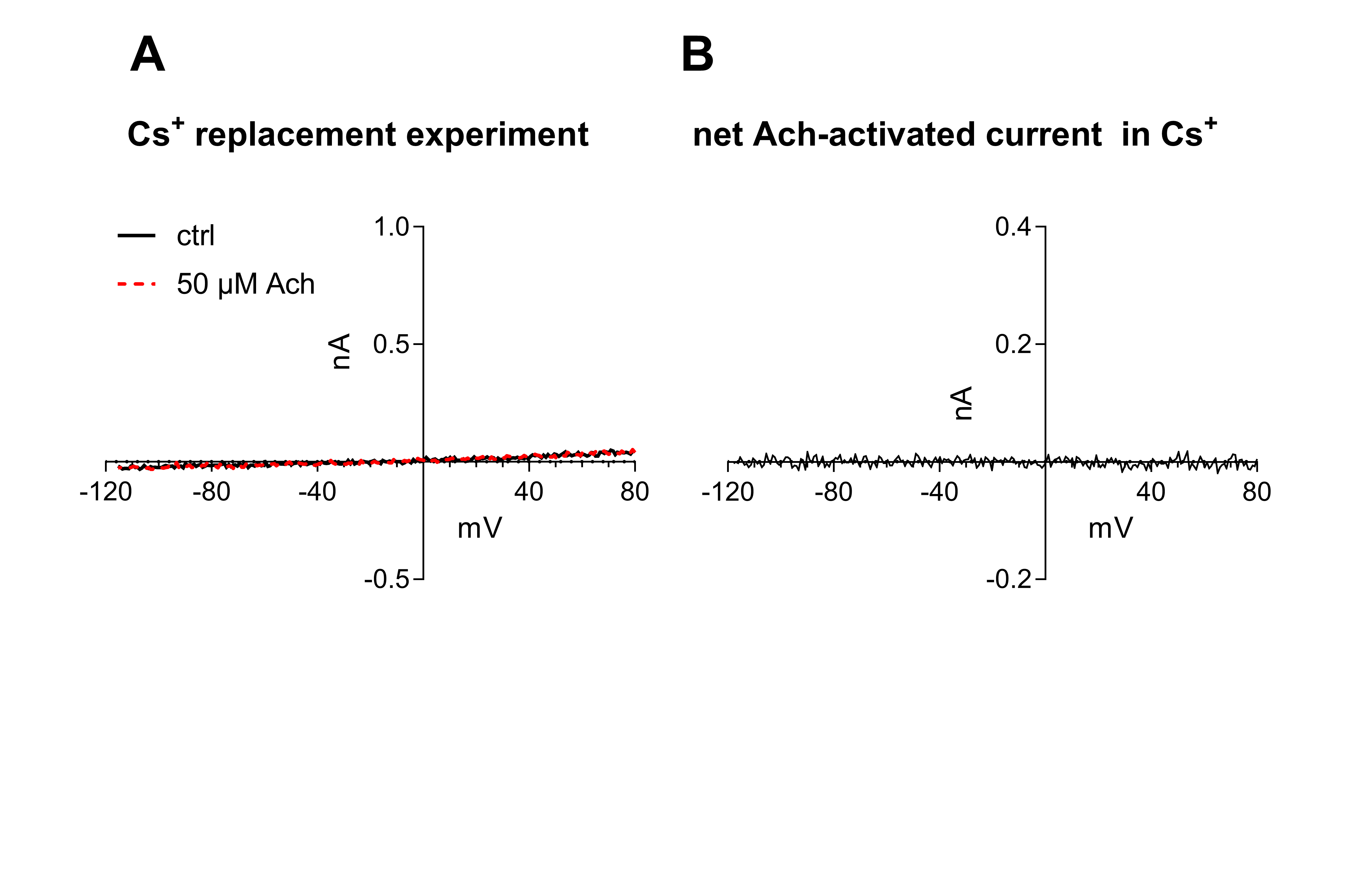
**

**Figure S1**. **Ach effect on ramp-evoked currents is prevented by Cs+ replacement of K+ ions.** A) Original patch clamp current traces, recorded by replacing intra- and extracellular K+ with equimolar Cs+, in a representative hfNBM cell, where a voltage ramp protocol (-120/+80 mV, 800 ms) was applied before (ctrl) and after 50 µM acetylcholine (Ach) application. Note the lack of Ach effect on ramp-evoked currents in this experimental condition. B. Ach-sensitive current, obtained by subtraction of the control ramp from that recorded in Ach, is abolished by Cs+ replacement.

- 1. **Supplementary Figure 2**


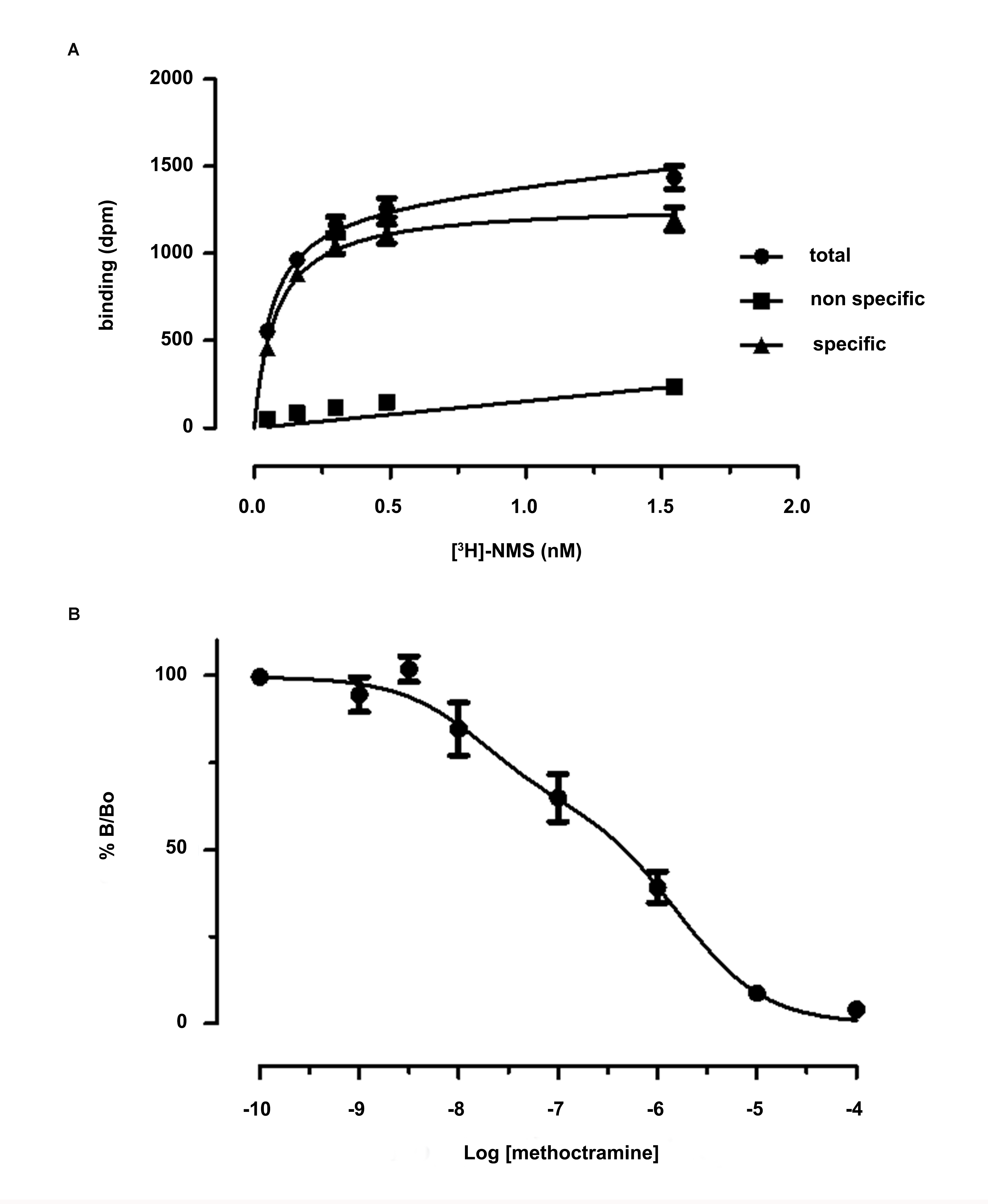


**Figure S2. Muscarinic receptor binding to membranes from hfNBMs.**

A) Representative saturation curves of the muscarinic receptor binding at increasing concentrations of the radioligand [^3^H]NMS are shown as total (black circles), specific (black triangles) and non-specific (black squares) binding with each data point showing the mean ± SEM of three independent experiments performed in duplicate. Calculated averages from all experiments (n=3) are described in the main text. The total binding increased progressively at increasing concentration of [^3^H]NMS. The non-specific binding included binding of the radioligand to glass fiber filters, adsorption to the preparation, and dissolution in the membrane lipids and did not saturate as the concentration of radioligand was increased. The specific binding was calculated as the difference between the total binding and non-specific binding and showed the real concentration of receptors, with the binding being saturable at the higher concentrations of [^3^H]NMS. Data sets of total and nonspecific binding obtained from each saturation-binding assay were globally fitted using nonlinear regression equation in GraphPad Prism to obtain the Kd and Bmax parameters. B) Competition binding curves showing the effect of increasing concentration of methoctramine on the specific binding of [^3^H]-NMS to membranes from hfNBM cells. Specific binding was determined by subtracting the values obtained in the presence of 10 µM atropine from the total binding and used for the normalization of the data as 100%. Data points are the mean ± SEM of three independent experiments performed in duplicate. Data were analyzed by both one-site and two-site competition models to obtain the Ki values, and were compared with an F test to obtain optimal fit. The curve represents the result of the two site fits (p=0.0012).

**2.3. Supplementary Figure 3**

**
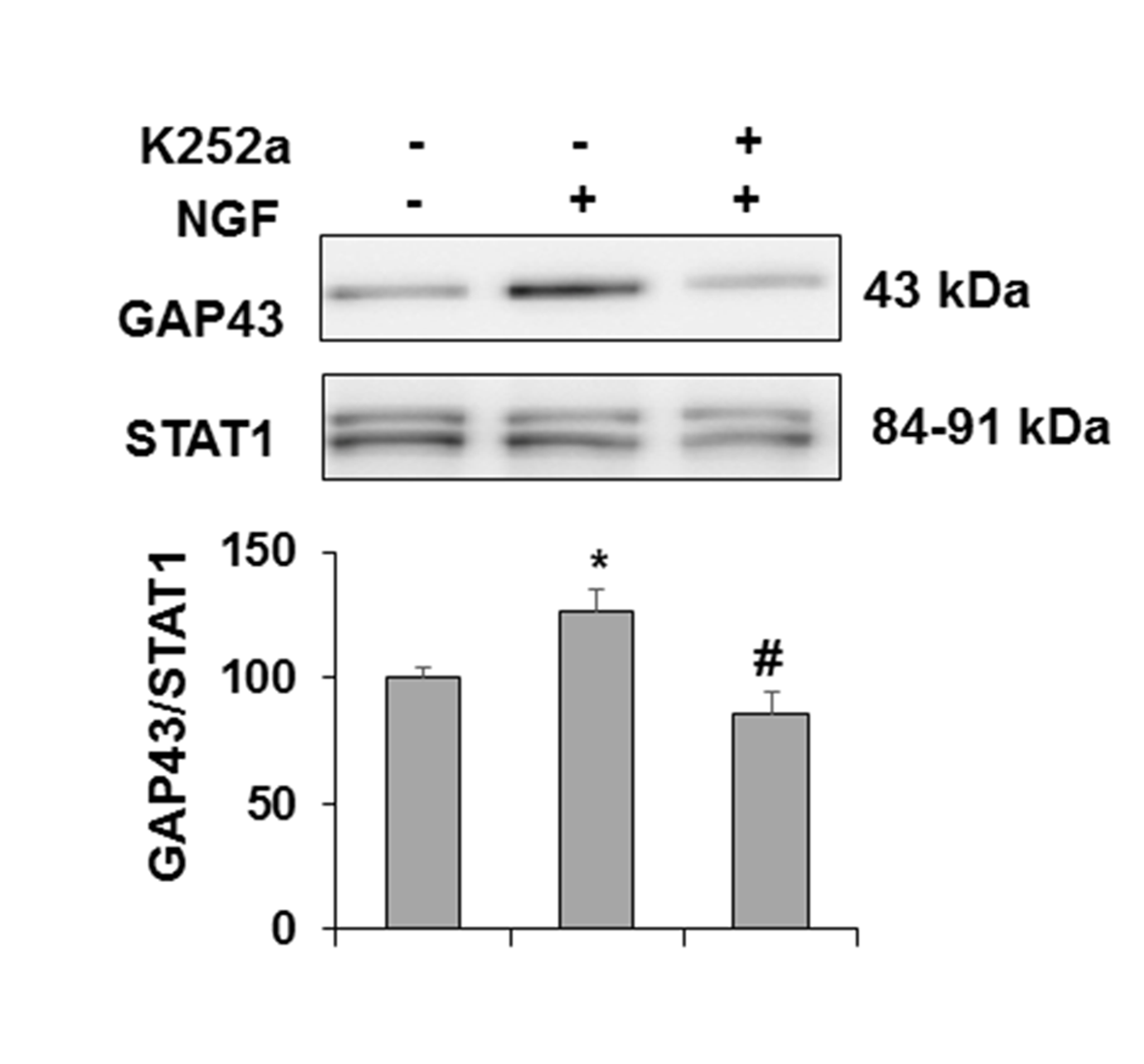
**

**Figure S3. NGF increases the expression of growth associated protein 43 (GAP43), a neurite outgrowth marker, in hfNBMs.**

Western blot analysis of (GAP43) expression in serum-starved cells treated or not (control) with 100 ng/ml NGF for 24h in presence or absence of 200 nM K252a; STAT1 expression was used as protein loading control. The bar graph shows the computer-assisted quantification of band intensity: data, normalized over STAT1 signal, are expressed as % of control and reported as mean ± SEM (*p<0.05 vs. control; #p<0.05 vs. NGF; n=3).

**2.4. Supplementary Figure 4**

**
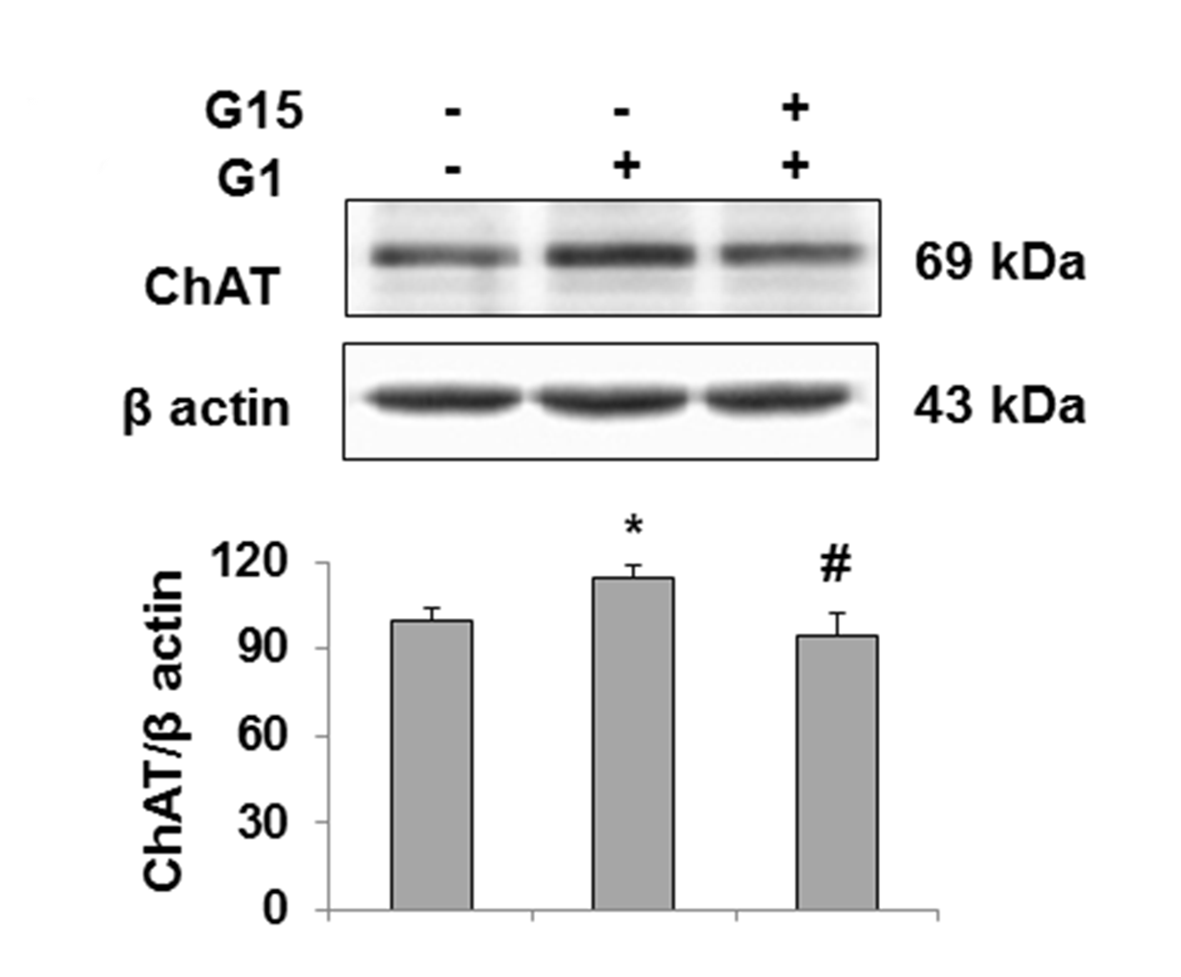
**

**Figure S4. GPR30 activation induces ChAT expression in hfNBMs.**

Western blot analysis of ChAT expression in serum-starved cells treated or not (control) with 100 nM G1 or with 100 nM G1+1µM G15 for 24h; β actin was used as protein loading control. The computer-assisted quantification of band intensity is represented in the bar graph: data, normalized over β actin signal, are expressed as % of control and reported as mean ± SEM (*p<0.05 vs. control; #p<0.05 vs. G1; n=3).

**2.5. Supplementary Table 1.**

**Gene expression assay ID (Life Technologies, Carlsbad, CA) for quantitative RT-PCR**

| **Gene** | **Assay ID number** |
| --- | --- |
| Nestin | Hs00707120_s1 |
| β-tubulin III | Hs00801390_s1 |
| Microtubule-associated protein 2 (MAP2) | Hs00234140_m1 |
| Glial fibrillary acidic protein (GFAP) | Hs00157674_m1 |
| Oligodendrocyte transcription factor 2 (Olig2) | Hs00377820_m1 |
| Choline acetyltransferase (ChAT) | Hs00252848_m1 |
| Acetylcholinesterase (AchE) | Hs00241307_m1 |
| Calbindin 1 (CALB1) | Hs00191821_m1 |
| CD271 | Hs00609976_m1 |
| Tropomyosin receptor kinase A (TRKA) | Hs01021011_m1 |
| Estrogen receptor α (ERα) | Hs01046818_m1 |
| Estrogen receptor β (ERβ) | Hs01100358_m1 |
| G protein-coupled estrogen receptor 1 (GPER/GPR30) | Hs00173506_m1 |
| Vesicular acetylcholine transporter (VAchT) | Hs00268179_s1 |
| 18S ribosomal subunit | Hs99999901_s1 |

**2.6. Supplementary Table 2**. Primer sequences for quantitative RT-PCR of nicotinic (CHRNA2-7; CHRNB2-4) or muscarinic (CHRM1-5) cholinergic receptor subunits.

| **Gene** |  | **Primer sequences (5’→3’)** |
| --- | --- | --- |
| CHRNA2 | F  R | TCATCTCCTGCCTCACTGTG  TCAGTGATGAGCAGCAGGAA |
| CHRNA3 | F  R | ATTCTTGAACCTGCTCCCCA  CAGATTTGAGAGCTCGGCAC |
| CHRNA4 | F  R | CGGCCATTTACAAGAGCTCC  CCACTCTCCCAGAAGTCCAG |
| CHRNA5 | F  R | TTCACACGCTTCCCAAACTG  CGCAGCTTCCAATGTGTTTC |
| CHRNA6 | F  R | GGGTGGTGGAAAATTCGGAG  CCATGGCCACGTATTTCCAG |
| CHRNA7 | F  R | CTGTTCGTTTCCCAGATGGC  TGTGGAATGTGGCGTCAAAG |
| CHRNB2 | F  R | AAGCCGCTCTTCTACACCAT  TTGGAGATGAGCAGCAGGAA |
| CHRNB3 | F  R | TGAAGGGGAACAGAAGGGAC  CAGGCAGGGGATGATGAGAA |
| CHRNB4 | F  R | CGACTTCATCATCAAGCGCA  TTGGAGATGAGCAGCAGGAA |
| CHRM1 | F  R | GGTTTCCTTTGTGCTCTGGG  GTAGAGCCTGCACATGACTG |
| CHRM2 | F  R | AATGCTGCTGTCACCTTTGG  CTCTTGCTGGCTCGGGATAT |
| CHRM3 | F  R | CAACCTCGCCTTTGTTTCCA  CGAAATGAGTGACGGTTCCC |
| CHRM4 | F  R | CTACTTCTGCGTCACCAAGC  CTTACCCACCACAAACTGCC |
| CHRM5 | F  R | AGTTTCTCTCTGAGCCCACC  TGGTCACAGAGTCAGAACCC |
